# Phosphatidylserine decarboxylase downregulation in uric acid‑induced hepatic mitochondrial dysfunction and apoptosis

**DOI:** 10.1002/mco2.336

**Published:** 2023-07-26

**Authors:** Ning Liu, Lei Huang, Hu Xu, Xinyu He, Xueqing He, Jun Cao, Wenjun Xu, Yaoxing Wang, Hongquan Wei, Sheng Wang, Hong Zheng, Shan Gao, Youzhi Xu, Wenjie Lu

**Affiliations:** ^1^ Basic Medical College Anhui Medical University Hefei Anhui China; ^2^ Zhejiang Provincial Laboratory of Life Sciences and Biomedicine, Key Laboratory of Growth Regulation and Transformation Research of Zhejiang Province, School of Life Sciences Westlake University Hangzhou Zhejiang China; ^3^ College of Life Sciences Zhejiang University Hangzhou Zhejiang China; ^4^ Institute of Biology Westlake Institute for Advanced Study Hangzhou Zhejiang Province China; ^5^ Center for Scientific Research Anhui Medical University Hefei Anhui China

**Keywords:** apoptosis, lipidomics, mitochondrial dysfunction, phosphatidylserine decarboxylase, STAT3

## Abstract

The molecular mechanisms underlying uric acid (UA)‐induced mitochondrial dysfunction and apoptosis have not yet been elucidated. Herein, we investigated underlying mechanisms of UA in the development of mitochondrial dysfunction and apoptosis. We analyzed blood samples of individuals with normal UA levels and patients with hyperuricemia. Results showed that patients with hyperuricemia had significantly elevated levels of alanine aminotransferase (ALT) and aspartate aminotransferase (AST) levels, which may indicate liver or mitochondrial damage in patients with hyperuricemia. Subsequently, lipidomic analysis of mouse liver tissue mitochondria and human liver L02 cell mitochondria was performed. Compared with control group levels, high UA increased mitochondrial phosphatidylserine (PS) and decreased mitochondrial phosphatidylethanolamine (PE) levels, whereas the expression of mitochondrial phosphatidylserine decarboxylase (PISD) that mediates PS and PE conversion was downregulated. High UA levels also inhibited signal transducer and activator of transcription 3 （STAT3） phosphorylation as well as mitochondrial respiration, while inducing apoptosis both in vivo and in vitro. Treatment with allopurinol, overexpression of PISD, and lyso‐PE (LPE) administration significantly attenuated the three above‐described effects in vitro. In conclusion, UA may induce mitochondrial dysfunction and apoptosis through mitochondrial PISD downregulation. This study provides a new perspective on liver damage caused by hyperuricemia.

## Graphical abstract

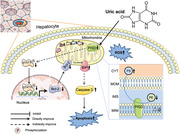

## 1 INTRODUCTION

The incidence of hyperuricemia has remained unchanged over the past decade, but its prevalence remains high (approximately 20% in U.S. adults and 14% in Chinese adults), as reported by previous studies.^1^, ^2^ Hyperuricemia is frequently associated with various health conditions, such as insulin resistance, dyslipidemia, metabolic syndrome,^3^, ^4^ kidney damage, hepatic steatosis, and cardiovascular disease.^5^, ^6^, ^7^ In a previous study, we found that high uric acid (UA) induced lipid disturbances mediated via lysophosphatidylcholine acyltransferase 3 (LPCAT3) upregulation in the liver, with hepatic damage observed in diet‐induced hyperuricemia model.^8^ UA has been considered an independent risk factor for fructose‐induced fatty liver.^9^ Allopurinol is a purine analog, whose administration can be used as an effective UA‐lowering therapy. Mechanistically, allopurinol inhibits xanthine oxidase, thereby reducing the formation of UA.^2^ Therefore, further investigation is crucial to understand the molecular mechanisms underlying the link between hyperuricemia and liver damage and the potential therapeutic benefits of classic drug allopurinol administration.

Liver damage is associated with mitochondrial dysfunction and apoptosis.^10^, ^11^ Several studies have analyzed the impact of hyperuricemia on organs and cells, such as inducing intracellular oxidative stress, particularly in human hepatoblastoma (HepG2) cells.^12^ In the kidney, oxidative stress induced by long‐term hyperuricemia or monosodium urate crystals influenced mitochondrial function.^13^, ^14^ In addition, UA promoted human proximal tubule cell apoptosis via oxidative stress and reduced nicotinamide adenine dinucleotide phosphate (NADPH) oxidase 4 activation.^15^ The endothelial dysfunction caused by UA was linked to changes in mitochondrial morphology and a reduction in the levels of intracellular adenosine triphosphate (ATP).^16^ Despite these findings, the precise mechanism of hyperuricemia‐induced hepatic mitochondrial dysfunction or apoptosis requires further investigation.

In our previous studies, we investigated the association between hyperuricemia and lipid metabolism. Our results indicated changes in cell membrane phospholipids and phosphorylation of signal transducer and activator of transcription 3 (p‐STAT3) expression.^8^ This led us to explore the potential impact of high UA on mitochondria phospholipids, which has not been studied in this context before. Our hypothesis was that UA might induce changes in mitochondrial lipids that, in turn, could trigger alterations in mitochondrial function, morphology, and apoptosis. These changes are interrelated with the previously studied STAT3 signaling pathway. Previous research has shown that STAT3 transcriptional activity regulates cell growth and stimulates the expression of antiapoptotic B‐cell lymphoma protein 2 (Bcl‐2).^17^, ^18^ Moreover, lipid homeostasis is critical for maintaining mitochondrial function. Inhibition of mitochondrial phospholipid biosynthesis enzymes can cause cellular respiratory dysfunction, leading to cell damage or death.^19^, ^20^ Conversely, elevated phospholipids in mitochondrial membranes enhance mitochondrial integrity, which can overcome chemotherapy‐induced tumor cell apoptosis.^21^ In this study, we aimed to explore changes in liver mitochondrial lipids caused by high UA and screen for key targets. We used liquid chromatography‐mass spectrometry (LC–MS)‐based lipidomics to identify abnormal lipids in liver tissue mitochondria, including phosphatidylserine (PS) and phosphatidylethanolamine (PE), in a mouse model of hyperuricemia. In addition, we investigated the underlying role of phosphatidylserine decarboxylase (PISD), which mediates the conversion of PS to PE in mitochondria.

PISD is expressed in a wide range of mouse tissues, including the heart, liver, lung, and testes.^22^ Intramitochondrial transport of PISD substrates involves lipid transfer from outer to inner membrane.^23^ Primarily, PISD triggers the rapid decarboxylation of PS to PE, which is the primary route of PS degradation in mammals, and also the sole pathway of mitochondrial PS catabolism.^24^ PISD is expressed in the mammalian mitochondrial intima and is a major source of PE in vivo.^25^, ^26^ PE is the second most abundant glycerophospholipid in eukaryotic cells. The importance of PE metabolism in mammals was recently recognized due to its association with various diseases.^27^ Moreover, studies have indicated that PE deficiency in mammalian mitochondria results in impaired oxidative phosphorylation (OXPHOS) and altered mitochondrial morphology.^28^ Therefore, PISD dysregulation inevitably leads to a change in PE content, affecting normal physiological function. Knocking down the *Pisd* gene compromised the integrity of mouse skeletal muscle fibers and mitochondria. Further, *Pisd* deletion in mice lead to fetal mortality and mitochondrial defects.^22^, ^29^ These results suggest a critical role of PISD in mitochondrial function.

Herein, we employed a mouse hyperuricemia model as well as normal human hepatic cells (L02) and HepG2 cells to study the effect of high UA levels on mitochondrial dysfunction and apoptosis. We determined changes in PS and PE following PISD downregulation, assessing the role of PE as a key regulator of hyperuricemia‐induced mitochondrial dysfunction and apoptosis. Our findings further indicate that PISD may be a critical enzyme in the process of mitochondrial lipid disorder caused by high UA levels. Furthermore, we explored the impact of PISD‐induced mitochondrial phospholipid disorder on STAT3 signal and apoptotic factor expression, including Bcl‐2, Bcl‐2 associated X (Bax), and cleaved caspase‐3. An in‐depth understanding of the effects of high UA levels on mitochondrial dysfunction and apoptosis and the underlying molecular mechanisms could offer valuable insights into the correlation between hyperuricemia and liver disease.

## 2 RESULTS

### 2.1 Hyperuricemia causes liver damage and hepatocyte apoptosis

**FIGURE 1:**
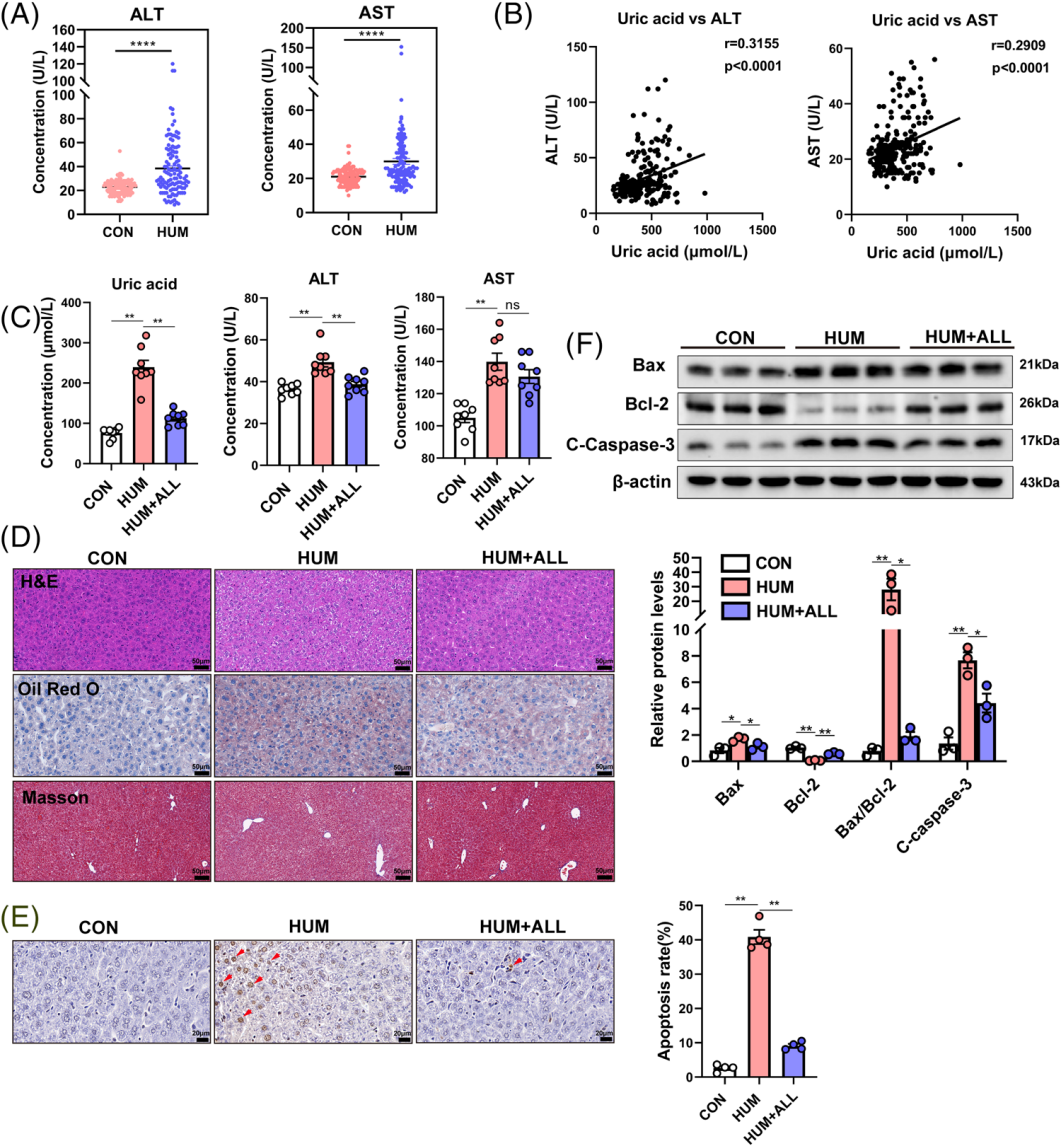
Changes in alanine aminotransferase (ALT) and aspartate aminotransferase (AST) of hyperuricemia patients and high uric acid (UA) causes liver damage and apoptosis in mice. (A) Serum ALT and AST levels in subjects with normal UA levels and patients with hyperuricemia. (B) Correlation analysis between UA and serum ALT or AST. (C) Serum UA, ALT, and AST in mice of three groups (CON group, HUM group, HUM+ALL group). (D) Mice liver histology as determined via hematoxylin and eosin (H&E), Oil Red O, and Masson's trichrome staining. (E) TUNEL staining was performed to detect cell apoptosis in the livers of mice. Apoptotic cells were identified by the presence of brown staining (red arrows). (F) Bcl‐2, Bax, and cleaved caspase‐3 protein levels in mouse livers were detected via western blotting. Data are presented as means ± SEM. *p < 0.05, **p < 0.01, ****p < 0.0001, ns indicates no significance. CON, control; HUM, mouse model of hyperuricemia; HUM+ALL allopurinol treatment group.

We collected blood samples of individuals with normal UA levels and patients with hyperuricemia. The results showed that the mean serum UA levels were 301.46 and 525.32 μmol/L in the normal group and hyperuricemia group, respectively. General patient characteristics and serum biochemical indexes are shown in Table S1. The hyperuricemia group exhibited significantly elevated levels of total cholesterol, triglycerides, and glucose, which suggests that hyperuricemia may be associated with obesity and lipid dysregulation. Further, patients with hyperuricemia had significantly higher serum alanine aminotransferase (ALT) and aspartate aminotransferase (AST) levels (Figure 1A), and the ratio of AST to ALT was decreased (Figure S1A), suggesting that hyperuricemia may be directly linked to marker enzymes of liver damage. Correlation analyses revealed a significant positive correlation between serum UA and both liver injury marker enzymes (AST and ALT) (Figure 1B).

We aimed to determine if UA plays a causative role in liver injury and mitochondrial damage. To achieve this, we established a mouse model of hyperuricemia (HUM group) by administering a hyperuricemia‐inducing diet (HID) comprising 2% oxonic acid potassium, 3% UA, and 95% standard chow to C57BL/6 mice for a duration of 8 weeks. In parallel, hyperuricemia was also established in treatment group (HUM+ALL group) mice, with allopurinol added to their water. The mice that were administered HID showed notably elevated serum levels of UA, ALT, and AST compared with the mice fed with a standard chow diet (SCD). Conversely, serum UA and ALT levels were significantly reduced in the treatment group relative to those in the HUM group (Figure 1C). Furthermore, the liver sections subjected to hematoxylin and eosin (H&E) and Oil Red O staining indicated augmented intrahepatic fat accumulation and liver injury in the HID‐fed mice (Figure 1D).

In addition, collagen deposition and liver fibrosis were not obvious in the livers of hyperuricemic mice compared with that observed in the control group (Figure 1D). Hepatocyte damage can lead to apoptosis, and mitochondria are the major apoptotic sensors.^30^ Terminal deoxynucleotidyl transferase dUTP nick‐end labeling (TUNEL) analysis revealed no apoptotic hepatocytes in control group samples, while the number of apoptotic hepatocytes was significantly increased in the HUM group relative to the HUM+ALL group (Figure 1E). Western blotting revealed a significant downregulation of Bcl‐2 in the HUM group, while the expression of Bax and cleaved caspase‐3 was significantly upregulated. Allopurinol treatment significantly alleviated these expression changes (Figure 1F). Taken together, these results demonstrated that hyperuricemia causes liver damage and hepatocyte apoptosis.

### 2.2 UA inhibits PISD in vivo

**FIGURE 2:**
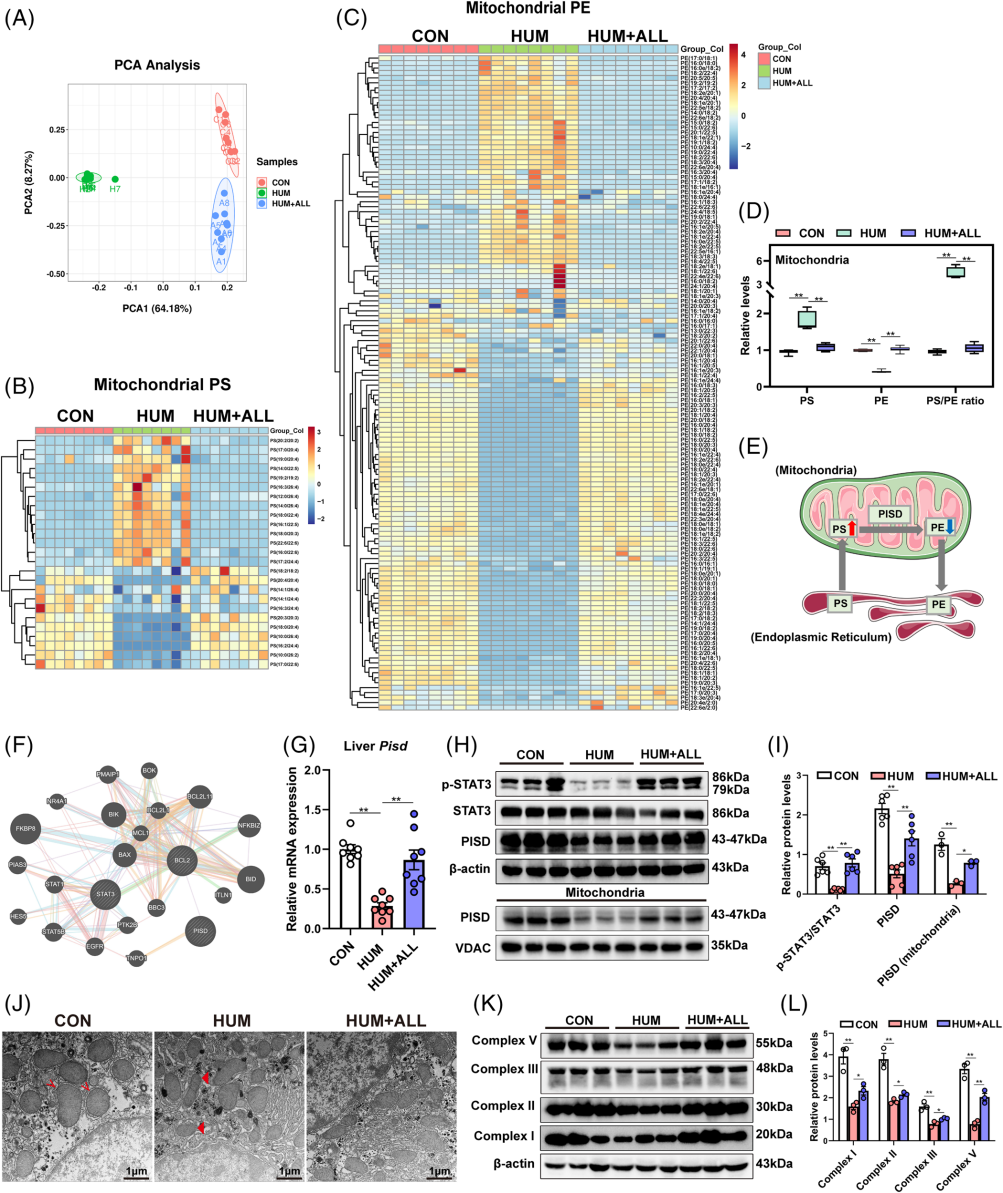
Uric acid suppressed the expression of phosphatidylserine decarboxylase (PISD) and the conversion of phosphatidylserine (PS) and phosphatidylethanolamine (PE) in vivo. (A) Principal component analysis (PCA) score plots based on mouse hepatic mitochondrial lipids identified via lipidomics. Dots represent each mouse of the CON group (red dots, *n* = 8), HUM group (green dots, *n* = 8), and HUM+ALL group (blue dots, *n* = 8). (B and C) Heatmap of the number of differentially abundant PSs (B) and PEs (C) in mouse liver mitochondria of three groups. (D) The quantification of total PS and total PE was performed in liver mitochondria of CON, HUM, and HUM+ALL mice, and the PS/PE ratio was calculated. (E) Mechanism of the PISD‐mediated conversion of PS to PE in mitochondria. (F) Bioinformatics analyses predicted an association between PISD and the STAT3/Bcl‐2 pathway (http://genemania.org/). (G) *Pisd* mRNA levels were determined via real‐time PCR of mouse liver tissues. (H) Western blotting was performed to detect total PISD and p‐STAT3 (Tyr705)/STAT3 levels and mitochondrial PISD in the livers of mice from the three groups. (I) Band intensities of each protein were quantified using ImageJ and normalized to that of the β‐actin/VDAC band. (J) Transmission electron microscopy images at an original magnification of 5000×. Representative electron microscopy representative images demonstrated marked alterations in the mitochondrial morphology of liver mitochondria from the three groups. Images revealed a disruption of the mitochondrial double membrane (thick arrowhead “◄”) in HUM group compared with well‐preserved double membrane (thin arrow “˂”) in CON group. (K and L) Hepatic levels of OXPHOS ETC complex proteins (complex I, II, III, V) were analyzed via Western blotting (K). Band intensities of each protein were quantified using ImageJ and normalized to that of the β‐actin band (L). Data are presented as means ± SEM. **p < 0.01, *p < 0.05. CON, control; HUM, mouse model of hyperuricemia; HUM+ALL allopurinol treatment group.

To investigate the possible mechanisms behind UA‐induced liver malfunctioning, apoptosis, and lipid metabolism disruption, LC–MS‐based lipidomics analysis was performed on liver mitochondria samples from control, hyperuricemia mice, and mice in the allopurinol‐treated group. A two‐component principal component analysis (PCA) score plot of LC–MS data was used to visualize the general variation in lipids among three groups. A clear separation between the control and hyperuricemia groups was observed, indicating a significant UA‐induced change in lipids (Figure 2A). The results showed that the proportion of various lipids was significantly different; PS and PE accounted for 1.8 and 9.5% of the total lipids, respectively (Figure S2A). Over 14 significantly upregulated PS (Figure 2B) and over 80 downregulated PE molecules were observed in hyperuricemic mouse liver mitochondria (Figure 2C). UA upregulated total PS and reduced total PE, with the PS/PE ratio increasing significantly, meanwhile, the ratio of PS to PE was significantly downregulated after allopurinol treatment (Figure 2D). In addition to PS and PE levels, the heatmap showed that the lipid changes of different subclasses in lysophosphatidylethanolamine (LPE), lysophosphatidylserine, lysophosphatidylcholine (LPC), cardiolipin (CL), phosphatidylinositol (PI), and phosphatidylglycerol (PG) were different in the hyperuricemia group (Figure S2B), and the statistical map showed that the total LPE, PI, and PG content were significantly increased, while the LPC, phosphatidylcholine (PC), CL, and sphingomyelin (SM) content were significantly decreased. Meanwhile, allopurinol played a therapeutic role in the allopurinol treatment group (Figure S2C). However, there were no significant changes in phosphatidylserine synthase 1 (*PTDSS1)*, which mediates the conversion of PC to PS, and phosphatidylserine synthase 2 (*PTDSS2)*, which mediates the conversion of PE to PS (Figure S2D).

The majority of cellular PE is formed via PISD‐catalyzed PS decarboxylation in the mitochondria.^25^ Thus, we speculated that UA altered PS and PE content within mitochondrial membranes through the action of PISD (Figure 2E). Bioinformatic predictions suggested an association between PISD and the STAT3/Bcl‐2 pathway (Figure 2F). Thus, we assessed PISD and STAT3 protein expression and found that both PISD and p‐STAT3/STAT3 were significantly downregulated in the HUM group. Consistent with the results of Western blotting, *Pisd* mRNA expression was downregulated in the HUM group and upregulated following allopurinol treatment (Figures 2G, H, and I). Further, PISD protein levels in hepatic mitochondria were also significantly decreased. Allopurinol treatment alleviated these expression changes (Figure 2H). Electron microscopy analysis indicated that the HUM group exhibited (i) a significant reduction in the number of cristae compared to the well‐maintained cristae in the control group, and (ii) disruption of the mitochondrial double membrane in contrast to the intact double membrane observed in the control group (Figure 2J). Allopurinol partially reversed the damage to mitochondrial structure (Figure 2J). As major sites of energy metabolism, mitochondria are closely related to apoptosis, lipid metabolism, and liver damage. Further, PISD plays a role in the inner mitochondrial membrane. We aimed to investigate if there was an impairment in mitochondrial respiration in the livers of hyperuricemia mice. Our analysis indicated a downregulation in the expression of mitochondrial OXPHOS proteins such as complex I (NADH:ubiquinone oxidoreductase), complex II (succinate‐coenzyme Q reductase), complex III (cytochrome b‐c1 complex subunit 2), and complex V (ATP synthase) in the hyperuricemia mouse livers. Allopurinol reversed UA‐induced mitochondrial respiratory chain dysregulation (Figures 2K and L). Taken together, UA may affect mitochondrial function and apoptosis through the PISD‐mediated PS–PE conversion.

### 2.3 UA triggers mitochondrial dysfunction and apoptosis in vitro

**FIGURE 3:**
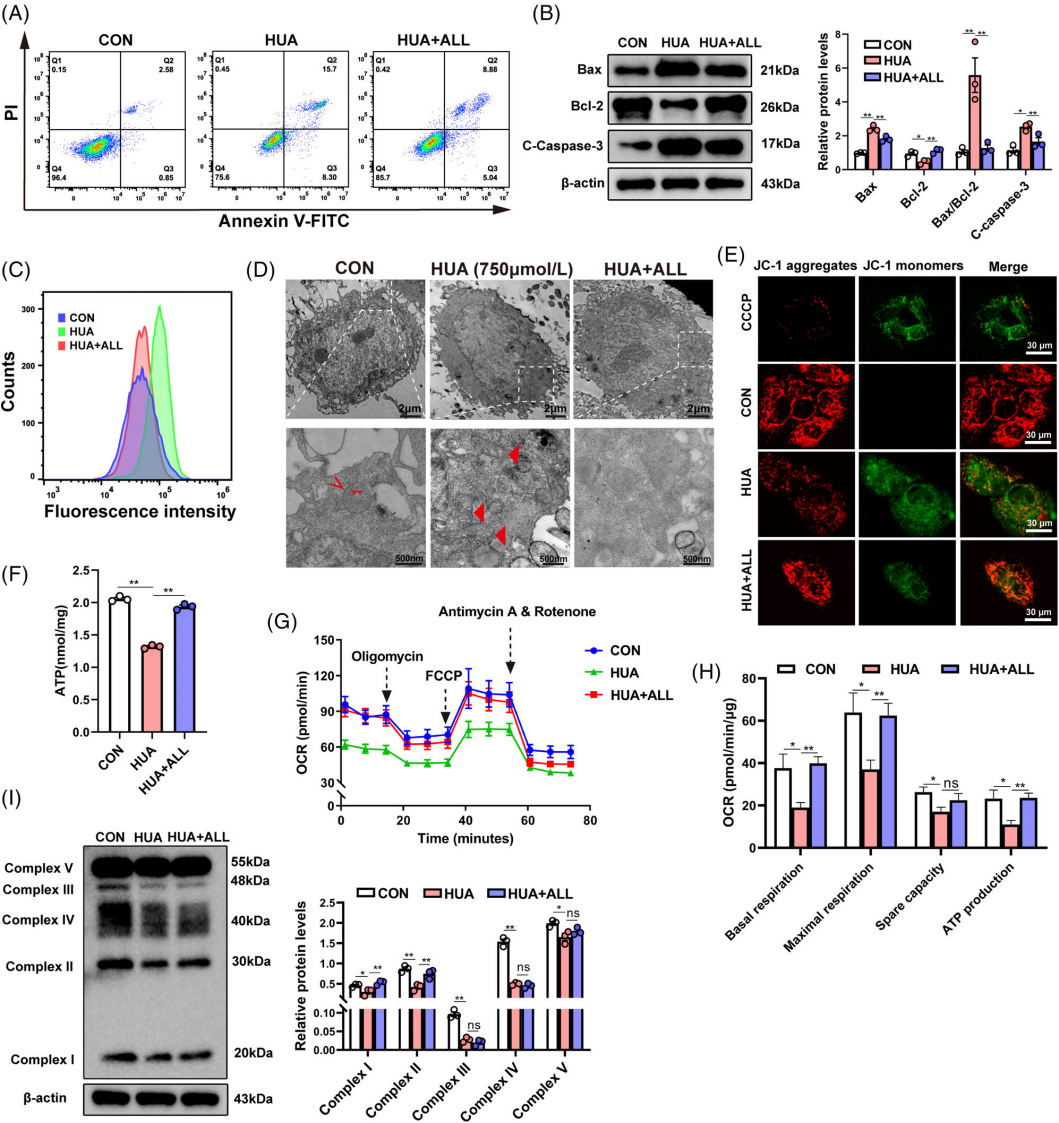
Uric acid (UA) triggers mitochondrial dysfunction and apoptosis in L02 cells. (A) UA‐induced apoptosis in L02 cells. Apoptosis was detected using Annexin V‐FITC and PI staining. (B) Bcl‐2, Bax, and cleaved caspase‐3 protein levels were detected via Western blotting. (C) The levels of ROS in L02 cells treated with UA (750 μmol/L) or allopurinol (100 μmol/L). (D) Transmission electron microscopy images at an original magnification of 3000× (top) and 10,000× (bottom). Representative electron microscopy images demonstrated marked alterations in the mitochondrial morphology of L02 cells incubated with 750 μmol/L UA for 48 h. Higher magnification (10,000×) revealed a disruption of the mitochondrial double membrane (thick arrowhead “◄”) in UA‐treated cells compared with well‐preserved cristae (thin arrowhead “▸”) and a double membrane (thin arrow “˂”) in control cells. (E) Changes in mitochondrial membrane potential (MMP) in L02 cells treated with UA or allopurinol were determined based on JC‐1 fluorescence. (F) ATP content in the three groups. (G) Mitochondrial oxygen consumption rate (OCR) measurements were performed with a Seahorse metabolic analyzer. Oligomycin (1.5 μM), FCCP (1 μM), and rotenone (0.5 μM) in addition to antimycin (0.5 μM) were sequentially added to L02 cells treated with or without UA (750 μmol/L) and allopurinol (100 μmol/L). (H) Quantitative analysis of mitochondrial function parameters (basal respiration, maximal respiration, spare capacity, and ATP production) was shown in the bar charts (*n* = 5). (I) OXPHOS protein expression (complex I–V) in L02 cells stimulated with high UA with or without allopurinol were analyzed via Western blotting. Data are presented as means ± SEM. **p < 0.01, *p < 0.05, ns indicates no significance.

In order to substantiate whether high UA is capable of inducing mitochondrial dysfunction and apoptosis through the PISD‐driven PS–PE pathway and STAT3/Bcl‐2 pathway, we utilized L02 and HepG2 cells. We conducted flow cytometry analysis using Annexin V‐propidium iodide (PI) staining on L02 cells that experienced UA (750 μmol/L) exposure for 48 h. Our findings established a significant upregulation in apoptosis brought about by high UA (Figure 3A). Western blotting revealed a significant downregulation of antiapoptotic Bcl‐2 in the high UA (HUA) group, while the expression of proapoptotic Bax and cleaved caspase‐3 was significantly upregulated. Consistent with the in vivo results, allopurinol alleviated UA‐induced apoptosis (Figure 3B). Mitochondria serve as a crucial source of reactive oxygen species (ROS), especially under stressful conditions.^31^ Therefore, the amount of ROS generated under different conditions was determined, revealing that UA significantly enhanced ROS production in L02 cells, while allopurinol had a certain inhibitory effect (Figure 3C).

To further investigate the effect of UA on mitochondrial function, we conducted a series of experiments in L02 cells. High levels of UA (750 μmol/L) considerably altered mitochondrial morphology, as determined via electron microscopy. High magnification (10,000×) revealed a noticeable decrease in the number of cristae, disruption of the mitochondrial double membrane, and the presence of a small number of vacuoles in UA‐treated cells relative to the considerably well‐preserved cristae and double membrane in control cells. Allopurinol reversed damage to the mitochondrial structure (Figure 3D). A noteworthy event in the early phases of apoptosis is the decrease in mitochondrial membrane potential (MMP; ∆*Ψ*m). Changeover of JC‐1 from red (JC‐1 aggregates) to green fluorescence (JC‐1 monomers) signifies a reduction in membrane potential. Pretreatment with high UA decreased MMP, while allopurinol treatment enhanced it (Figure 3E). UA suppressed both MMP and ATP production (Figures 3E and F). To better understand how UA modulates mitochondrial function in vitro, the mitochondrial oxidative capacity of L02 cells was determined using the Seahorse XF Cell Mito Stress Test under UA stimulation with or without allopurinol (Figure 3G). Our findings indicated that the oxygen consumption rate (OCR) of mitochondria in L02 cells significantly decreased due to high UA stimulation relative to the control cells. This significant deterioration in mitochondrial dysfunction was demonstrated by a reduction in basal respiration, maximal respiration, spare capacity, and ATP production (Figure 3H). Allopurinol had a certain therapeutic effect on mitochondrial function, as suggested by an increase in all four aforementioned parameters (Figure 3H). In addition, we discovered that the expression of mitochondrial OXPHOS proteins (complex I–V) decreased in L02 cells treated with high UA concentrations. Further, allopurinol treatment upregulated complex I and complex II expression relative to those in HUA cells (Figure 3I).

Consistent with results obtained using L02 cells, an increase in HepG2 cell apoptosis was observed after stimulation with UA (Figure S3A). UA activated apoptotic pathways (Figure S3B), enhanced ROS production (Figure S3C), damaged the mitochondrial structure (Figure S3D), decreased MMP (Figure S3E), suppressed ATP production (Figure S3F), disturbed mitochondrial function (Figures S3G and H), and downregulated OXPHOS proteins (complex I–V) (Figure S3I). Consistent with previous results, allopurinol alleviated UA‐induced mitochondrial dysfunction and apoptosis in HepG2 cells. Taken together, our data showed that UA may trigger mitochondrial dysfunction and apoptosis in vitro.

### 2.4 PISD expression is inhibited by high UA concentrations in vitro

**FIGURE 4:**
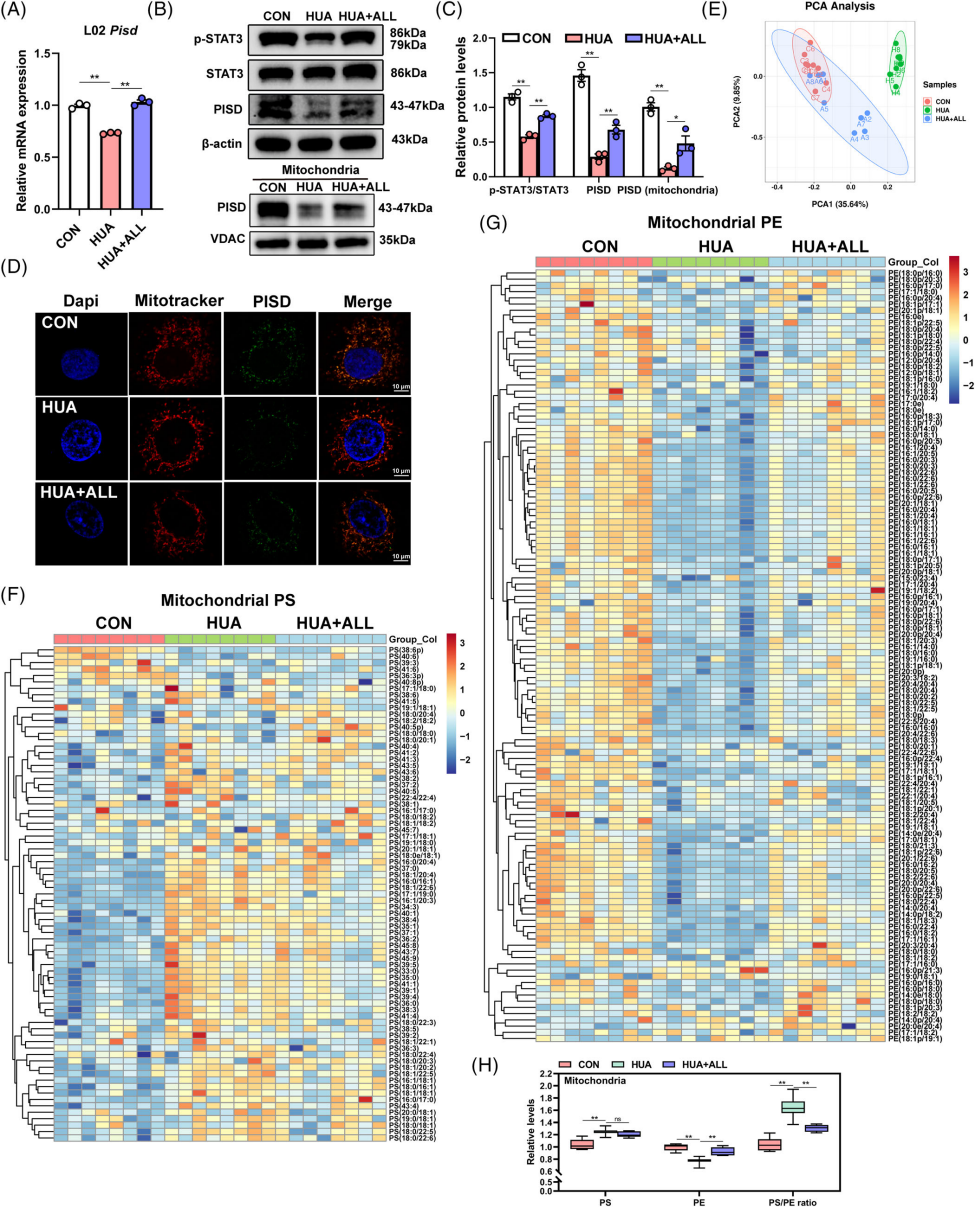
The expression of phosphatidylserine decarboxylase (PISD) in L02 cells is inhibited by high uric acid (UA) concentration. (A) *Pisd* mRNA levels were determined via real‐time PCR in L02 cells of three groups (CON group, HUA group, HUA+ALL group). (B and C) Representative Western blots showing total protein expression of PISD, STAT3, p‐STAT3 (Tyr705), as well as PISD protein levels in mitochondria (B). Band intensities of each protein were quantified using ImageJ and normalized to that of β‐actin or VDAC (C). (D) Representative confocal microscopy image of DAPI (blue), PISD (green), and the mitochondrial marker (red) in control cells as well as cells exposed to UA (750 μmol/L) and allopurinol (100 μmol/L) (×630). (E) Principal component analysis (PCA) score plots of lipids identified via lipidomics. Dots represent each sample of the different groups, including the CON group (red dots, *n* = 8), HUA group (green dots, *n* = 8), and HUA+ALL group (blue dots, *n* = 8). (F and G) Heatmap of differentially abundant phosphatidylserine (PS) (F) and phosphatidylethanolamine (PE) (G) in mitochondria isolated from L02 cells based on lipidomics analysis. The color of each section is proportional to the significance of change in lipid abundance. Positive correlations (yellow/red, upregulated) and negative correlations (blue, downregulated) are shown. (H) The quantification of total PS and total PE in mitochondria isolated from L02 cells. The PS/PE ratio was determined in cells of all three groups. Data are presented as means ± SEM. **p < 0.01, *p < 0.05. CON, control; HUM, mouse model of hyperuricemia; HUM+ALL allopurinol treatment group.

Consistent with in vivo results, we observed significant decreases in PISD and p‐STAT3/STAT3, both at the mRNA and protein levels, in UA‐stimulated L02 cells (Figures 4A–C). Mitochondrial PISD expression was also significantly decreased (Figures 4B and C). Allopurinol treatment significantly alleviated these expression changes. Likewise, significant downregulation of PISD at the mRNA (Figure S4A), protein (Figure S4B), and mitochondrial‐specific protein (Figure S4C) levels was observed in HepG2 cells. Meanwhile, under the stimulation of different concentrations of UA (0, 300, 500, 750, and 1000 μmol/L), we found that with the increase of UA concentration, the protein expression of PISD decreased gradually (Figure S5A). PISD and mitochondrial markers colocalized, indicating that the PISD protein was localized in the mitochondria of normal L02 cells, as observed via confocal microscopy. After UA stimulation for 48 h, mitochondrial PISD expression was significantly downregulated, with allopurinol blocking this reduction (Figure 4D).

We sought to confirm whether UA‐induced mitochondrial dysregulation is mediated via PISD‐catalyzed PS–PE conversion in mitochondria. LC–MS‐based lipidomics was performed on mitochondria isolated from control L02 cells (CON), HUA group, and treatment group (HUA+ALL) cells. Control and HUA cells were well separated in PCA plots, highlighting the difference between lipidomic profiles. Upon allopurinol treatment, lipidomic profiles shifted back to that of control cells, reflecting the compound's protective effect (Figure 4E). Consistent with in vivo results, PSs were significantly upregulated (Figure 4F), and PEs were significantly downregulated in the mitochondria of UA‐stimulated L02 cells (Figure 4G). Further, we found that UA upregulated total PS and reduced total PE within mitochondria, significantly increasing the PS/PE ratio (Figure 4H). Allopurinol significantly reversed changes in PE and PS abundance. In addition to PS and PE, the heatmap showed that the lipid changes of different subclasses in LPE, LPC, CL, PI, and PG were different in the hyperuricemia group (Figure S5B), and the statistical map showed that the total LPE, LPC, PC, CL, PI, PG, and SM content were significantly increased, meanwhile, allopurinol in the allopurinol treatment group played a certain therapeutic role (Figure S5C). These results demonstrated the UA‐mediated inhibition of PISD in vitro, with significant changes of PS and PE within the mitochondrial membrane.

### 2.5 PISD overexpression ameliorates UA‐induced mitochondrial dysfunction and apoptosis in L02 cells

**FIGURE 5:**
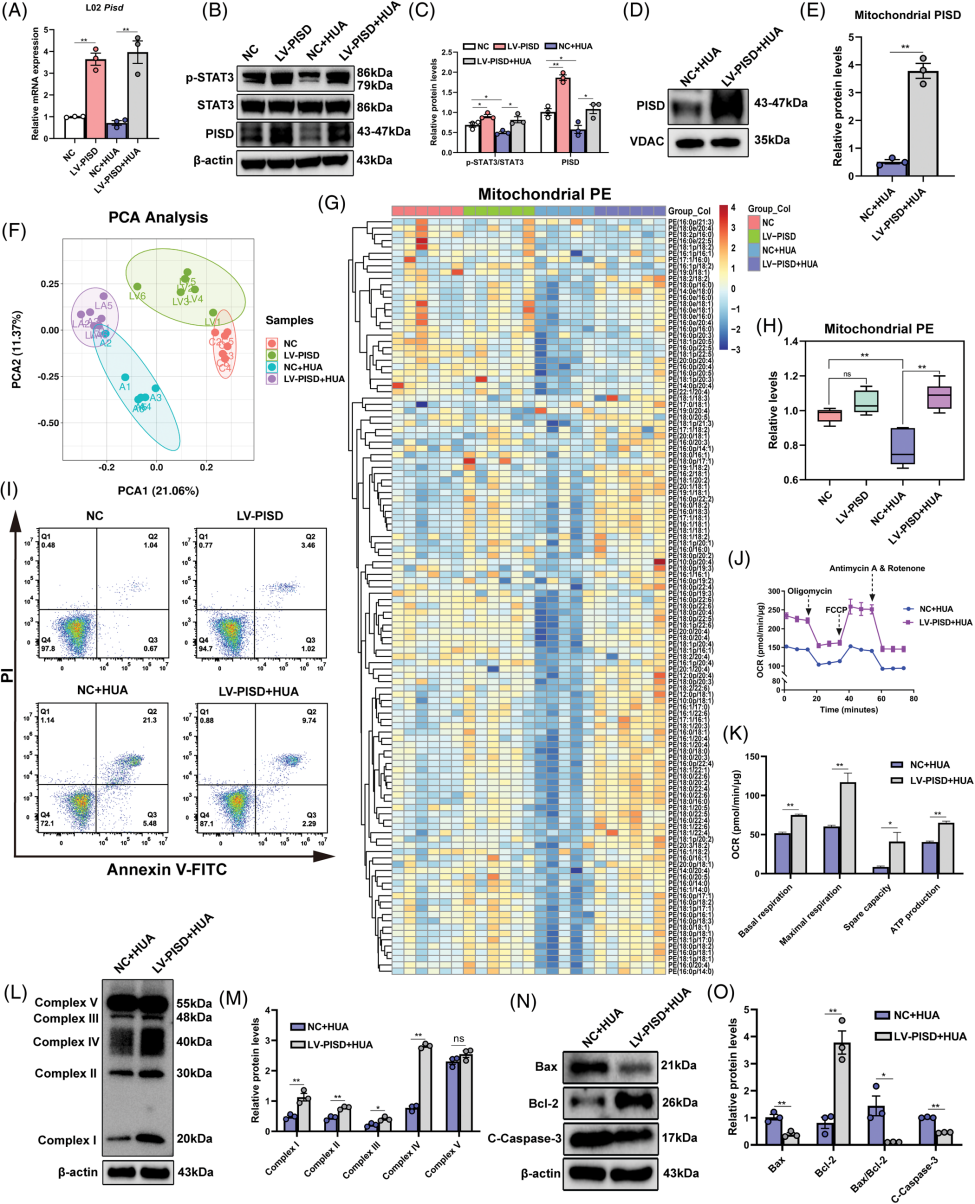
The overexpression of phosphatidylserine decarboxylase (PISD) ameliorates uric acid (UA)‐induced mitochondrial dysfunction and apoptosis in L02 cells. (A) *Pisd* mRNA levels were determined via real‐time PCR in L02 cells of four groups (NC group, LV‐PISD group, NC+HUA group, LV‐PISD+HUA group). (B and C) Representative Western blots showing protein expression of PISD, STAT3, and p‐STAT3 (Tyr705) in cells of the four groups. (D and E) Mitochondrial PISD protein levels in NC+HUA and LV‐PISD+HUA cells. (F) Principal component analysis (PCA) score plots based on lipids identified via lipidomics. Dots represent each sample of different groups, including the NC group (red dots, *n* = 6), LV‐PISD group (green dots, *n* = 6), NC+HUA group (blue dots, *n* = 5), and LV‐PISD+HUA group (purple dots, *n* = 6). (G and H) Heatmap of differentially abundant phosphatidylethanolamine (PE) in mitochondria isolated from L02 cells of four groups based on lipidomics. Positive correlations (yellow/red, upregulated) and negative correlations (blue, downregulated) are shown (G). Total PE in mitochondria of the NC+HUA and LV‐PISD+HUA cells was quantified (H). (I) Apoptosis was detected via Annexin V‐FITC and PI staining in the NC group, LV‐PISD group, NC+HUA group, and LV‐PISD+HUA group. (J) Mitochondrial oxygen consumption rate (OCR) measurements were performed using a Seahorse metabolic analyzer. Oligomycin (1.5 μM), FCCP (1 μM), and rotenone (0.5 μM) combined with antimycin (0.5 μM) were added sequentially to L02 cells in the NC+HUA and LV‐PISD+HUA group. (K) Quantitative analysis of mitochondrial function parameters (basal respiration, maximal respiration, spare capacity, and ATP production) is shown in the bar charts (*n* = 5). (L and M) OXPHOS protein expression (complex I–V) in L02 cells of the NC+HUA group and LV‐PISD+HUA group were analyzed via Western blotting. (N and O) Bcl‐2, Bax, and cleaved caspase‐3 protein levels of the NC+HUA and LV‐PISD+HUA group were detected via Western blotting. Data are means ± SEM. **p < 0.01, *p < 0.05. NC, negative control; LV, lentivirus; HUA, high UA.

Recently, several studies have highlighted the crucial role of PISD in the preservation of skeletal muscle mitochondrial integrity and muscle mass.^29^ As UA could induce mitochondrial damage and increase ROS production, we questioned whether UA induced mitochondrial dysfunction directly or indirectly via PISD. To assess this, PISD expression in L02 cells was selectively upregulated via lentivirus (LV) transfection (Figure 5A). Consistent with mRNA expression, we found significant increases in PISD and p‐STAT3/STAT3 protein levels in PISD‐overexpressing L02 cells, suggesting that PISD may be involved in the same pathway as STAT3, directly or indirectly affecting its phosphorylation (Figures 5B and C). A significant increase in PISD protein was observed in UA‐stimulated cell mitochondria under PISD overexpression (LV‐PISD+HUA) when compared to control cells (NC+HUA) (Figures 5D and E).

To further clarify the effect of PISD overexpression on mitochondrial function in the high UA model, lipidomics was performed on mitochondria isolated from L02 cells of the control, LV‐PISD, NC+HUA, and LV‐PISD+HUA groups. The groups were separated in the two‐dimensional PCA plot (Figure 5F), with variation in differentially abundant PEs. Over 120 PEs were significantly upregulated in mitochondria of the LV‐PISD+HUA compared with the NC+HUA group (Figure 5G). Total PE was significantly increased in the mitochondria of LV‐PISD+HUA group cells when compared with NC+HUA group (Figure 5H). Taken together, PISD overexpression could prevent the UA‐induced decrease in PE.

Consistent with lipidomics results, overexpression of PISD significantly reduced apoptosis and enhanced MMP and decreased ROS production (Figures 5I and S6A, B). Mitochondrial function was significantly improved in the LV‐PISD+HUA group (Figure 5J). The OCR of mitochondria in the LV‐PISD+HUA group was significantly higher than those in the NC+HUA group, as indicated by increased basal respiration, maximal respiration, and spare capacity, as well as higher ATP production (Figure 5K). The expression of mitochondrial OXPHOS proteins (complex I–V) was significantly increased in the LV‐PISD+HUA group (Figures 5L and M). Meanwhile, UA decreased the expression of proapoptotic proteins Bax and cleaved caspase‐3 in parallel to antiapoptotic protein Bcl‐2 upregulation (Figures 5N and O).

### 2.6 Restoration of UA‐induced mitochondrial dysfunction and apoptosis with exogenous lyso‐PE

**FIGURE 6:**
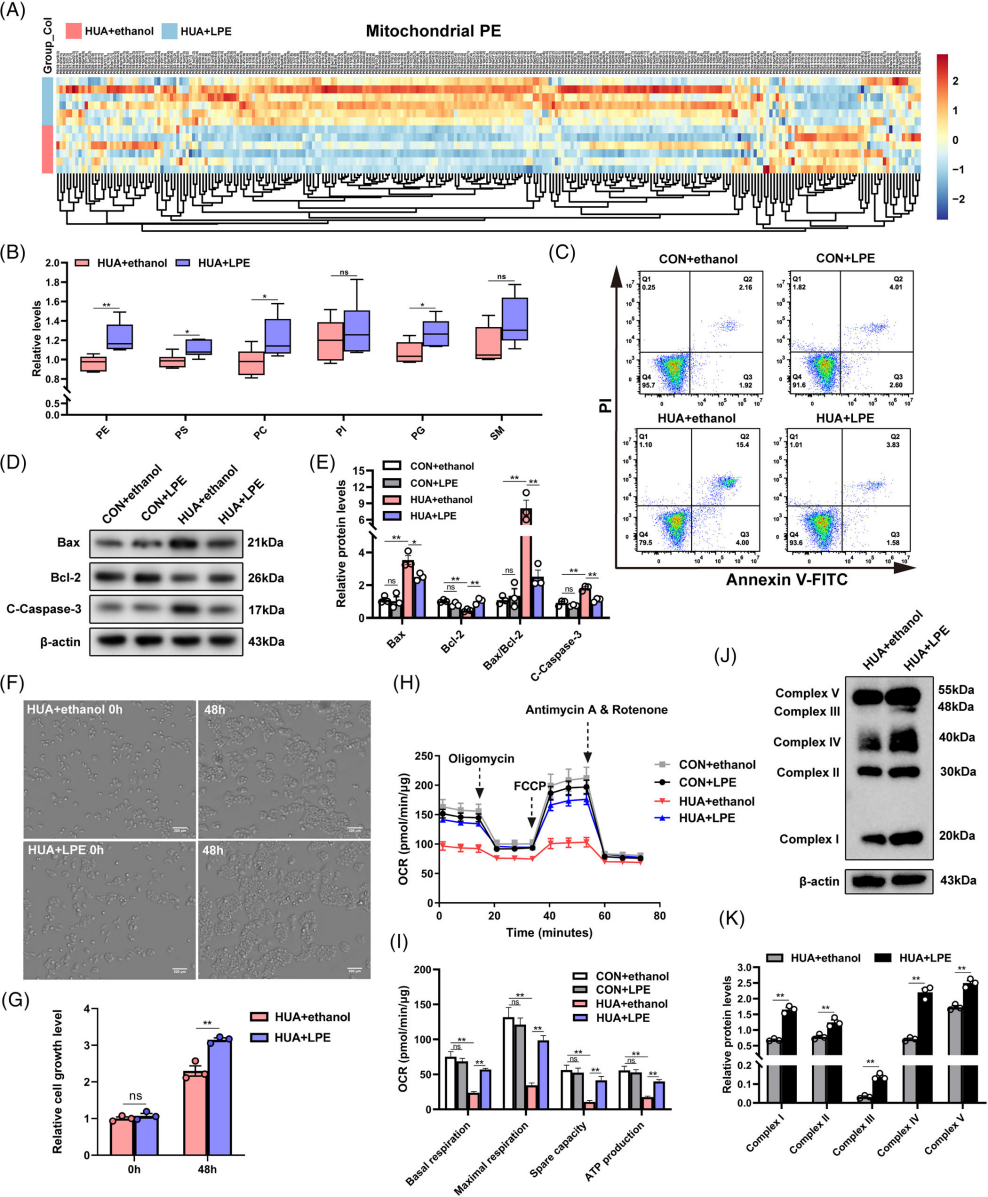
Lyso‐phosphatidylethanolamine (PE) supplementation of L02 cells treated with high uric acid (UA). (A) Heatmap of differential PEs in mitochondria isolated from L02 cells between HUA+ethanol and HUA+LPE group based on lipidomics (*n* = 6). Positive correlations (yellow/red, upregulated) and negative correlations (blue, downregulated) are shown. (B) Total PE, PS, PC, PI, PG, and SM were quantified in mitochondria of HUA+ethanol and HUA+LPE group cells. (C) Apoptosis was detected via Annexin V‐FITC and PI staining in the four (CON+ethanol, CON+LPE, HUA+ethanol, and HUA+LPE) groups. (D and E) Bcl‐2, Bax, and cleaved caspase‐3 protein levels of the CON+ethanol, CON+LPE, HUA+ethanol, and HUA+LPE groups were detected via Western blotting. (F and G) Following 48 h of incubation with 100 μM lyso‐PE, the confluence of UA‐treated L02 cells increased based on analysis performed using the Cell discoverer live‑cell imaging and analysis system. Scale bar, 200 μm. (H) Mitochondrial oxygen consumption rate (OCR) measurements were performed using a Seahorse metabolic analyzer. Oligomycin (1.5 μM), FCCP (1 μM), and rotenone (0.5 μM) combined with antimycin (0.5 μM) were added sequentially to L02 cells of the CON+ethanol group, CON+LPE group, HUA+ethanol group, and HUA+LPE group. (I) Quantitative analyses of mitochondrial function parameters (basal respiration, maximal respiration, spare capacity, and ATP production) are shown in the bar charts (*n* = 8). (J and K) OXPHOS protein expression (complex I–V) in L02 cells of the HUA+ethanol and HUA+LPE group were analyzed by Western blotting. Data are presented as means ± SEM. **p < 0.01, *p < 0.05, ns indicates no significance. LPE, lysophosphatidylethanolamine; HUA, high UA; PS, phosphatidylserine; PC, phosphatidylcholine; PI, phosphatidylinositol; PG, phosphatidylglycerol; SM, sphingomyelin.

To validate that the mitochondrial defects caused by UA in L02 cells were precisely due to the reduction of mitochondrial PE (mtPE), we administered 100 μM lyso‐PE treatment to the cells, which is preferentially converted to mtPE.^32^ Lyso‐PE supplementation of cells treated with UA for 48 h increased mtPE (Figure 6A). Lipidomics analysis on mitochondria isolated from L02 cells of the HUA+ethanol and HUA+LPE groups revealed significantly upregulated PEs in the HUA+LPE group mitochondria compared with those of HUA+ethanol group. Total PE was significantly increased in the mitochondria of the HUA+LPE group (Figure 6B). However, some other lipids (PS, PC, and PG) were also slightly up‐regulated in the mitochondria of the HUA+LPE group (Figure 6B). Lyso‐PE supplementation significantly suppressed apoptosis in the HUA+LPE group relative to controls (HUA+ethanol group). In the absence of UA stimulation, there was no significant difference in apoptosis between the LPE group and the ethanol group (Figure 6C). Bcl‐2 was upregulated, while Bax and cleaved caspase‐3 were downregulated after treatment with lyso‐PE for 48 h (Figures 6D and E). In addition, lyso‐PE supplementation also significantly improved the membrane potential drop under UA stimulation (Figure S6C).

Live‑cell imaging and analysis revealed that lyso‐PE supplementation enhanced cell growth (Figures 6F and G, and Supplementary Movie S1, 2). The OCR in the HUA+LPE group was significantly higher than in the HUA+ethanol group, as indicated by increased basal respiration, maximal respiration, and spare capacity, as well as higher ATP production (Figures 6H and I). Likewise, mitochondrial function was assessed in HepG2 cells of the HUA+ethanol and HUA+LPE groups, revealing increased basal respiration and increased maximal respiration in the latter group (Figures S7A and B). Furthermore, the expression of OXPHOS protein increased in HUA+LPE group (Figures 6J and K). Thus, replenishment of mitochondrial PE (mtPE) in UA‐treated L02 cells markedly improved cell growth, ATP production, apoptosis, and mitochondrial function.

## 3 DISCUSSION

The liver plays a vital role in numerous physiological processes, such as blood volume regulation, endocrine control of growth signaling pathways, and lipid and cholesterol homeostasis,^33^ and hepatic cell death promotes liver disease progression.^34^, ^35^ However, the mechanisms behind some of the impacts of hyperuricemia on the liver are still unknown. Mitochondria play crucial roles in regulating hepatic redox balance, lipid metabolism, and cell death.^36^, ^37^, ^38^ Furthermore, the electron transport chain (ETC) is the primary subcellular source of ROS that can damage mitochondrial proteins, lipids, and DNA.^39^ Mitochondrial phospholipids have a vital role in preserving mitochondrial function,^20^ whereas both the cell and mitochondrial lipid composition are known to influence the sensitivity of cancer cells to pharmacological inhibition of electron transport chain complex I and play an integral part in maintaining ROS homeostasis.^40^ Therefore, we propose that hyperuricemia‐induced liver damage may result from mitochondrial phospholipid dysfunction, which has not been investigated extensively. To investigate changes in mitochondrial lipid homeostasis caused by high UA levels, we conducted mitochondrial lipidomics research on hyperuricemia mice and L02 cells following high UA stimulation.

We found that the ratio of PS/PE was increased, and that UA altered the content of PS and PE in mitochondria mediated by PISD. However, the increased PS/PE ratio may also suggest an increase in the conversion of reactive PE to PS, which may be catalyzed by PS synthase. Therefore, we used qRT‐PCR to determine the enzyme *PTDSS1* (conversion of PC to PS) and the enzyme *PTDSS2* (conversion of PE to PS)^41^ and found that the expression of PS synthase did not change significantly in the liver of hyperuricemia mice, which may further reflect the importance of PISD. Meanwhile, many other mitochondrial lipids (including PC, CL, PI, and PG) also underwent significant changes such as the reduction of CL content, which may be the focus of future work. The deficiency of CL can disrupt the assembly of ATP synthase, which will directly affect the formation of mitochondrial inner membrane cristae.^42^ Animal studies found that some pathological states related to apoptosis, such as aging and ischemia–reperfusion, are accompanied by a decrease in CL content, and apoptosis occurs with a reduction in total CL content.^43^, ^44^ The effects of these lipids were consistent with our conclusions.

In our study, PISD protein expression and PE content were significantly decreased after UA stimulation. Related research has shown that inhibiting PISD‐driven mtPE synthesis in mice profoundly altered mitochondrial morphology and was embryonic lethal.^28^ Several studies have emphasized the critical role of PISD in maintaining muscle mass,^29^ and changes in PE abundance were associated with Alzheimer's disease, Parkinson's disease,^27^ glucose metabolism,^45^ and liver disease.^46^ However, the effects of UA on PISD function and PE metabolism remain unclear.

Previously, research has indicated that the predominant mtPE synthesis in CHO cells^47^ and yeast^48^ is attributed to the mitochondrial PISD, indicating that the import of PE produced from CDP‐ethanolamine may not adequately contribute to maintaining normal mtPE levels. This inadequacy may clarify the reason why a partial loss of PISD cannot be compensated for by the CDP‐ethanolamine pathway. PISD was previously regarded only as a PE producer. A more complex role has been suggested for this enzyme recently. In addition to the importance of PE in regulating the dynamic membrane structure, increasing evidence has shown that PE is responsible for protein stabilization, especially within mitochondrial membranes.^25^ Reduced mtPE content has been linked to compromised respiratory capacity, ETC enzymatic activities, and ATP synthesis. Inhibition of the ETC is typically associated with reduced MMP. Both chronic (PSB‐2 cells) and acute (Pisd KD cells) mtPE deficiencies in mammalian cells cause impairments in cell growth, mitochondrial morphology, respiratory ability, ATP production, and ETC activity.^28^ Crucially, in L02 cells, UA‐induced mitochondrial defects could be largely remedied by normalizing mtPE levels through lyso‐PE supplementation, leading to restored cell growth and mitochondrial respiratory function.

In addition to mitochondrial lipid disturbances, we have also observed increased ROS and apoptosis in the hyperuricemia model, which is consistent with previous literature.^15^, ^49^ Lipid peroxidation of phospholipid bilayers can promote mitochondrial apoptosis and complex molecular signaling pathways that regulate apoptosis.^50^ Additionally, we observed that the phosphorylation of STAT3 significantly decreased under the influence of UA, consistent with our previous article, and STAT3 is closely related to lipid homeostasis. STAT3 has been reported to be a transcriptional activator of Bcl‐2, inducing its expression by transferring into the nucleus to activate Bcl‐2.^51^, ^52^ In osteosarcoma cells, it has been suggested that STAT3 mediates apoptosis by inhibiting Bcl‐2 after apatinib treatment.^53^ We aim to connect these closely related pathways and propose that while PISD may not directly bind to specific proteins such as STAT3, BAX, or Bcl‐2, it acts upstream to affect these phenotypic changes by regulating mitochondrial lipid homeostasis.

In terms of results, PISD overexpression and lyso‐PE supplementation improved mitochondrial function, leading to a decrease in apoptosis and enhanced cell proliferation. We observed that high UA levels inhibited STAT3 phosphorylation and Bcl‐2 expression in mouse livers and L02 cells. Furthermore, as downstream molecules of Bcl‐2 in the apoptosis pathway,^54^ we observed elevated proapoptotic BAX and cleaved caspase‐3 levels in the hyperuricemia model. Overexpression of PISD significantly attenuated UA‐induced p‐STAT3 and Bcl‐2 suppression in L02 cells, suggesting that high UA levels may affect the p‐STAT3/Bcl‐2 apoptosis pathway via PISD downregulation.

A limitation of the current study is that we did not ascertain exactly how UA regulates PISD expression, and the direct relationship between PISD and STAT3 also remains unclear. Meanwhile, we will continue to explore the effect of UA on other lipids or other enzymes. The dysbiosis of gut microbiota has been demonstrated to be an important factor in the development of hyperuricemia.^55^ The increase of UA in the bloodstream acts as an important compensatory mechanism that is excreted through the intestines, directly influencing the gut microenvironment, promoting changes in bacterial growth and metabolism, and leading to intestinal dysfunction.^56^, ^57^ These intestinal dysfunctions may also be closely related to liver damage, which will be investigated in our subsequent studies.

**FIGURE 7:**
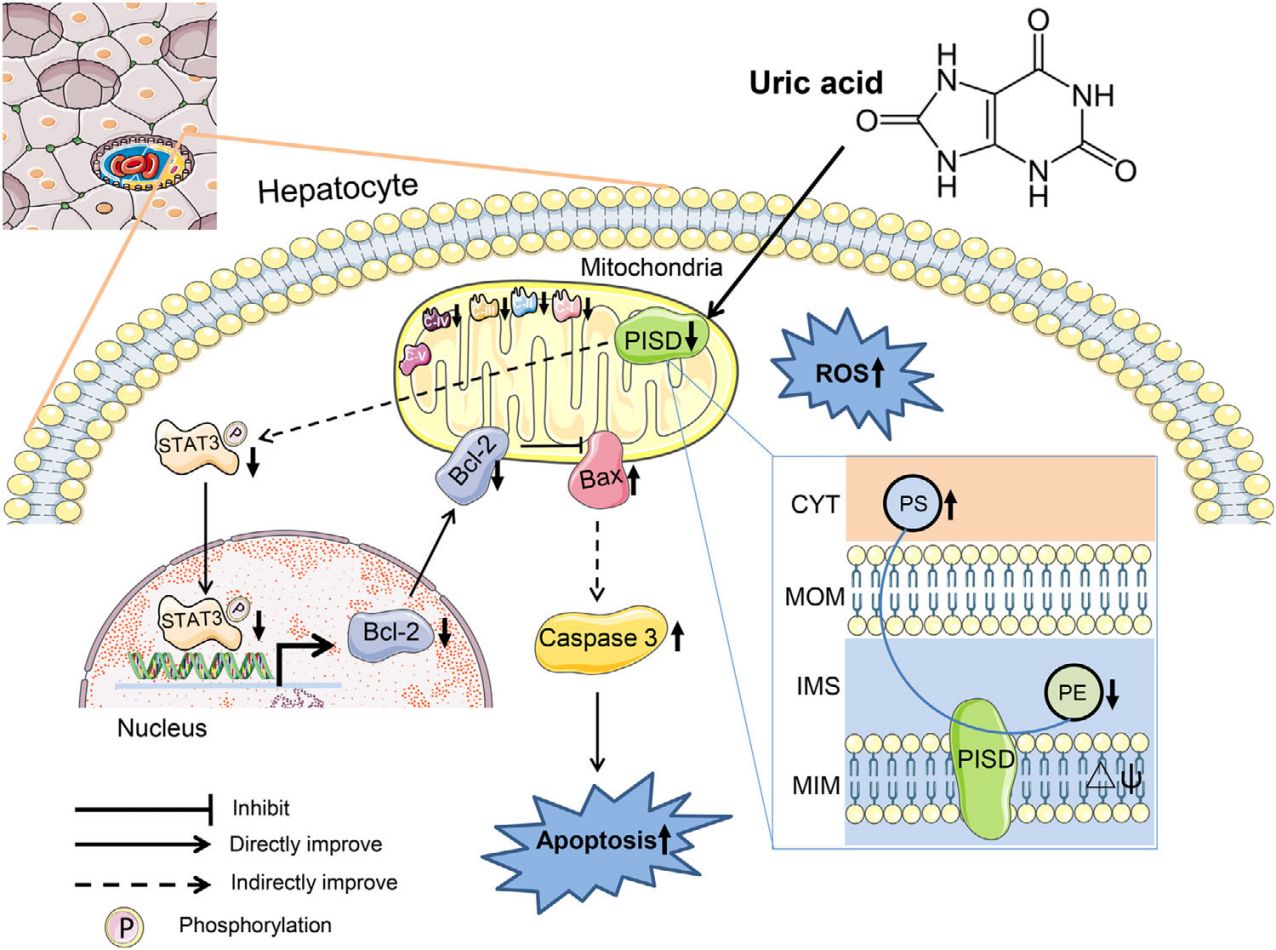
Possible mechanism underlying the regulatory effect of uric acid (UA) on mitochondrial dysfunction and apoptosis. After entering hepatocytes, UA inhibits the conversion of PS to PE mediated by downregulating PISD in mitochondria. The phosphorylation of STAT3 is reduced, and apoptotic signaling (Bcl‐2, Bax, and cleaved caspase‐3) is activated. PISD, phosphatidylserine decarboxylase; PS, phosphatidylserine; PE, phosphatidylethanolamine; STAT3, signal transducer and activator of transcription 3; IMS, mitochondrial intermembrane space; MIM, mitochondrial inner membrane; MOM, mitochondrial outer membrane; Δ*ψ*, membrane potential; C I–V, mitochondrial respiratory chain complex I–V.

In conclusion, our study sheds light on the critical role of mitochondria in hyperuricemia by identifying the role of UA in hepatic mitochondrial phospholipid homeostasis through PISD. High UA levels lead to a reduction in PE content via PISD suppression, resulting in mitochondrial dysfunction, the activation of the STAT3/Bcl‐2 apoptosis pathway, and liver injury (as illustrated in Figure 7). These results offer a new perspective on the pathogenesis of hyperuricemia‐associated liver injury.

## 4 MATERIALS AND METHODS

### 4.1 Clinical subjects and samples

A total of 240 samples from both hyperuricemia patients (40 females and 80 males) and individuals with normal UA levels (40 females and 80 males) were confirmed clinically at the First Affiliated Hospital of Anhui Medical University, and basic clinical information was recorded. Blood samples were then collected after a 12‐h fasting period and serum was separated by centrifugation at 1000×*g* for 10 min at 4°C. The corresponding kits for ALT and AST were used to analyze the serum using a Hitachi 7020 automatic biochemistry analyzer. The remaining serum was stored at −80°C. This study received approval from the Biomedical Research Ethics Committee of Anhui Medical University and written informed consent was acquired from all patients enrolled in the study.

### 4.2 Animal models

Male C57BL/6J mice (6−8 weeks old, 18−22 g) were procured from Jinan Pengyue Experimental Animal Breeding Co., Ltd. The mice were placed in clear plastic cages, with five individuals per cage, in a specific pathogen‐free and temperature‐controlled environment with a 12‐h light/dark cycle. Food and water were available ad libitum. Prior to the experiment, all mice were acclimatized for a week. The mice were divided randomly into three groups and given a SCD (*n* = 8), HID (*n* = 8), or HID combined with allopurinol (120 mg/L) (A8803; Sigma–Aldrich) (provided normal drinking water for the first 30 days, and then given water with allopurinol for the next 30 days) (*n* = 8). The HID was made up of 3% UA (U2625; Sigma–Aldrich), 2% oxonic acid potassium (156124; Sigma–Aldrich), and 95% SCD.^7^ After 60 days, the mice were dissected. The animal experiments were approved by the Experimental Animal Ethics Committee of Anhui Medical University.

### 4.3 Mouse serum biochemical analysis

Mouse serum samples from three groups were collected for UA, ALT, and AST determination using a commercial kit following the instructions provided by the manufacturer (UA: C012‐2‐1; ALT: C009‐2‐1; AST: C010‐2‐1) (Jiancheng Institute of Biotechnology). Serum was separated through centrifugation (1000×*g* for 15 min) and directly analyzed.

### 4.4 Histological analysis

Liver tissues were extracted and immersed in 4% paraformaldehyde solution for more than 24 h. The tissues were then embedded in paraffin wax and sliced into 4 μm serial sections for standard H&E staining and Masson's trichrome staining. To determine hepatic fat accumulation, frozen liver sections measuring 8 μm were stained sequentially with Oil Red O and hematoxylin using standard techniques. All staining procedures were conducted by Servicebio (http://www.servicebio.com/).

TUNEL staining was performed to detect apoptosis following the DAB (SA‐HRP) TUNEL cell apoptosis detection kit (G1507; Servicebio), and captured using an automated digital slide scanner (The PANNORAMIC MIDI II; 3DHISTECH Ltd.). Apoptotic cells were identified by brown staining, manually counted within the selected area, and quantified based on the number of TUNEL positive cells.

### 4.5 Cell culture

Human hepatoblastoma cell line (HepG2) and normal human hepatic cell line (L02) were obtained from the Chinese Academy of Science. The cells were cultured in DMEM (SH30243.01; Hyclone) and RPMI 1640 (SH30027.01; Hyclone) supplemented with 10% FBS and 1% penicillin/streptomycin in a 5% CO_2_ at 37°C. To investigate the effect of UA on mitochondrial dysfunction and apoptosis, the cells were treated with 750 μmol/L UA for either 24 or 48 h. In experiments incorporating allopurinol, the cells were preincubated with allopurinol (100 μmol/L) (Cat# T0692; Targetmol) for 8 h prior to exposure to high UA levels.

The LV for overexpressing PISD was procured from Genechem. PISD LV and negative control (NC) LV were incubated with L02 cells overnight. After 72 h, puromycin was used to select stable transfected cell lines for further mechanism research.

To supplement the cells with LPE, an ethanolic solution of 100 μM LPE (18:1, 846725p; Avanti Polar Lipids) was added to the cells and they were incubated for 96 h. An equivalent amount of ethanol was added to cells that were not supplemented with LPE.

### 4.6 RNA isolation and real‐time PCR

Total RNA was extracted from liver tissues of mice and cells using the TRIzol (CW0580; CoWin Biosciences). Total RNA (2 μg) was reverse transcribed to cDNA using the reverse transcription kit (K1622; Thermo Fisher Scientific), following these synthesis conditions: 5 min at 25°C, 60 min at 42°C, and 15 min at 70°C. We measured the mRNA expression levels using qRT‐PCR with the CFX96 Real‐Time PCR Detection System (Bio‐Rad Laboratories). Each gene's mRNA expression level was normalized to GAPDH. The primer sequences used for qRT‐PCR analyses are listed in Supplementary Table S2.

### 4.7 Mitochondria isolation

To conduct Western blot analysis, collected cells and tissues were treated with mitochondrial separation reagent (Beyotime), and homogenized on ice 20−30 times. The cells and tissues were then centrifuged at 600×*g* and 4°C for 5 min, and the supernatant was transferred to another centrifuge tube and centrifuged again at 11,000×*g* and 4°C for 10 min. After removing the supernatant carefully, we were able to isolate the precipitate, which contained the mitochondria.

For Mass spectrometry‐based lipidomics analysis, mitochondria were extracted according to the relevant protocol.^58^ Briefly, tissues and collected cells were transferred to the chilled homogenizer tube. A sufficient amount of homogenization buffer (210 mM mannitol, 70 mM sucrose, 5 mM Tris–HCl [pH 7.5], and 1 mM EDTA [pH 7.5]) was added using a Dounce homogenizer to prepare a 1:10 (w/v) homogenate. The homogenate was transferred to a centrifuge tube and centrifuged at 1300×*g* for 10 min to pellet unbroken cells, nuclei, plasma membranes, and fibers of connective tissue. After the supernatant was transferred to a clean tube, the pellet was resuspended in half the original volume of homogenization buffer and centrifuged again at 1300×*g* for 10 min. The two supernatants were pooled and centrifuged at 600×*g* for 10 min. The supernatant was then centrifuged at 11,000×*g* for 15 min to pellet mitochondria.

### 4.8 Western blot analysis

Proteins were extracted from cells, liver tissues, and mitochondria using RIPA buffer (Beyotime) with protease and phosphatase inhibitors (Beyotime). Separated by 8−12% SDS‐PAGE, an equal amount of protein was transferred to PVDF membranes (Millipore, Inc.). After blocking with 5% nonfat dry milk in TBST, primary antibodies were added and left to incubate overnight. These antibodies include anti‐PISD (sc‐390070, 1:500; Santa Cruz, CA), anti‐p‐STAT3 (9145, 1:2000; Cell Signaling), anti‐STAT3 (9139, 1:1000; Cell Signaling), anti‐COX IV (4850, 1:1000; Cell Signaling), anti‐Bcl‐2 (AF6139, 1:1000; Affinity), anti‐Bax (AF0120, 1:1000; Affinity), anti‐cleaved caspase‐3 (AF7022, 1:1000; Affinity), anti‐actin (AF7018, 1:6000; Affinity), and OXPHOS rodent WB antibody cocktail (45‐8099, 1:1000; Thermo Fisher Scientific). The blot was then further incubated with HRP‐conjugated secondary antibodies, either goat anti‐rabbit (S0001; Affinity) or goat anti‐mouse (S0002; Affinity). Finally, proteins were visualized with an enhanced chemiluminescence kit (Bio‐Rad).

### 4.9 Immunofluorescence and confocal microscopy

The LSM 880 multiphoton confocal microscope from Carl Zeiss in Germany was used to image the cells, and Zeiss software was used to analyze the images.^59^ L02 cells were cultured in specialized confocal cell dishes. To visualize mitochondria following treatment, the cells were stained with 100 nM Mito‐Tracker Red CMXRos (Molecular Probes) and incubated for 30 min. Post incubation, the cells were washed with PBS and fixed in 4% paraformaldehyde for 20 min at room temperature. After blocking with 5% goat serum albumin (Beyotime) and 0.1% Triton X‐100 (Sigma) for 30 min at room temperature, the cells were incubated overnight at 4°C with a primary antibody specific for PISD (1:100); followed by incubation with a secondary antibody (1:200, Elabscience) for 1 h at room temperature. The cells were finally washed with 1×PBS and their nuclei were stained with DAPI for 10 min (Solarbio). Visualization of the cells was carried out using a confocal microscope from Carl Zeiss in Germany.

### 4.10 Determination of the MMP with JC‐1 and ATP levels

After being exposed to UA and allopurinol, both L02 and HepG2 cells were washed twice with PBS. JC‐1 staining solution (Beyotime) was then added and the cells were incubated. Following staining, they were washed twice with a buffer solution before images were acquired using laser confocal fluorescence microscopy from LSM 880 (ZEISS). The ratio of green fluorescence intensity to red fluorescence intensity was used as an indicator of MMP.

Cell lysis was carried out using lysis buffer, and then, centrifuged at 12,000×*g* for 5 min at 4°C. The supernatant obtained was used to measure ATP levels. A dilution buffer containing luciferase (Beyotime) was immediately mixed with 50 μL of the supernatant, following which the mixture was incubated at room temperature for 3 min. Luminance (relative light units) was measured using an automatic microplate reader (Thermo Scientific). The ATP concentration was calculated based on a standard curve.

### 4.11 ROS and apoptosis measurement

To analyze the ROS generation, L02 cells and HepG2 cells were separately cultured in culture flasks and treated with different substances. The cells were collected and then suspended in DCFH‐DA (10 mmol/L) (Beyotime) with a cell concentration of 1−2 × 10^6^/mL. They were incubated at 37°C for 20 min, following which they were washed three times using serum‐free cell culture medium to completely eliminate DCFH‐DA. The cellular ROS level was determined using a flow cytometer (CytoFLEX).

The corresponding kit (E‐CK‐A211; Elabscience) was used to measure apoptosis. Cells were digested using trypsin that did not contain EDTA, and 1−5 × 10^5^ cells were collected and centrifuged (1000×*g*, 5 min) at 4°C,. Finally, 100 μL of 1× binding buffer was added to resuspend cells. Five microliters of Annexin V‐FITC and 10 μL of PI Staining Solution was added to the cells and gently mixed. The cells were incubated in darkness at room temperature for 10−15 min. Flow cytometry analysis was performed within 1 h (using CytoFLEX, Beckman).

### 4.12 Electron microscopy

Freshly dissected liver tissue was cut into longitudinal sections ∼2 mm in diameter and ∼2 mm in length and the cells were trypsinized to collect the pellet. Tissues and collected cell pellet were stored in a fixation mixture (4% paraformaldehyde and 2% glutaraldehyde) (Servicebio) at 4°C overnight. The resulting samples were sectioned into ultra‐thin slices, which were treated with uranyl acetate and osmium tetroxide for staining. Finally, transmission electron microscopy images were captured using microscope (Talos L120C; Thermo Fisher Scientific).

### 4.13 Mitochondrial respiration measurements

Mitochondrial OCR was assessed with the Seahorse XFe96 Analyzer (Seahorse Bioscience, Agilent). To perform the analysis, L02 cells (8000/well) and HepG2 cells (5000/well) were seeded into 96 wells of Seahorse XF96 cell culture microplates (Seahorse Bioscience). The cells were treated as required, and then the XF Assay Medium (Seahorse Bioscience), supplemented with 1 mM pyruvate, 2 mM glutamine, and 10 mM D‐glucose, was added to the wells. The cell culture microplates were then placed in a 37°C non‐CO_2_ incubator for 1 h, following which the Seahorse Bioscience XF96 Extracellular Flux Analyzer was used to measure the OCR. OCR was measured under four different conditions: (1) basal levels, without any additives; (2) with oligomycin (1.5 μM) to inhibit ATP synthase; (3) with FCCP (1 μM), a mitochondrial uncoupler, inducing maximal respiration; and (4) with rotenone/antimycin A (0.5 μM), a mitochondrial poison and complex I inhibitor, to end the reaction. The results were plotted using the Seahorse software.

### 4.14 Live‑cell imaging and analysis system

A live‑cell imaging and analysis system was used to observe cell proliferation (Celldiscoverer 7.0; Carle Zeiss). After stimulating L02 cells with UA and LPE, we placed the cell plate into the living cell growth detection instrument. Three points were selected from each well, and the system captured an image of each point every 2 h for 48 h, without moving the cell plate during that time. The relative cell proliferation ratio was calculated by comparing the cell confluence (%) of cells treated with or without LPE at the same time point.

### 4.15 Mass spectrometry‐based mitochondrial lipidomics

#### 4.15.1 Metabolites extraction

Mitochondria isolated from cells and livers of mice were transferred with 250 μL of water. After vortexing for 30 s, the samples were frozen and thawed with liquid nitrogen thrice. We then took out 50 μL of the mixture for protein determination and added 480 μL of MTBE: MEOH (5:1) to the remaining samples. After a 30‐s vortex, the samples were sonicated for 10 min in an ice‐water bath, followed by incubation at −40°C for 1 h and centrifugation at 900×*g* for 15 min at 4°C. Three hundred microliters of supernatant was transferred to a fresh tube and dried it in a vacuum concentrator at 37°C. The dried samples were reconstituted in a 50% methanol in dichloromethane solution by sonication on ice for 10 min and centrifuged at 16,200×*g* for 15 min at 4°C. Seventy‐five microliters of supernatant was transferred to a fresh glass vial for LC/MS analysis, and prepared a quality control sample by mixing an equal aliquot (20 μL) of the supernatants from all the samples.

#### 4.15.2 LC–MS/MS analysis

LC–MS/MS analyses were performed by Shanghai Biotree Biotech Co. Ltd using an UHPLC system (Vanquish; Thermo Fisher Scientific) with a UPLC HSS T3 column (2.1 mm × 100 mm, 1.8 μm) coupled to Q Exactive HFX mass spectrometer (Orbitrap MS; Thermo). The mobile phase A contained 40% water, 60% acetonitrile, and 10 mmol/L ammonium formate, whereas mobile phase B consisted of 10% acetonitrile, 90% isopropanol, and 50 mL of 10 mmol/L ammonium formate for every 1000 mL pour in. The analysis parameters for eluting gradient were as follows: 0–1.0 min, 40% B; 1.0–12.0 min, 40–100% B; 12.0–13.5 min, 100% B; 13.5–13.7 min, 100–40% B; 13.7–18.0 min, 40% B. The column temperature was set to 55°C, whereas the auto‐sampler temperature was 4°C. The injection volumes for positive and negative modes were 2 μL each.

The ESI source conditions were set as following: sheath gas flow rate as 30 Arb, Aux gas flow rate as 10 Arb, capillary temperature 350°C, full MS resolution as 120,000, MS/MS resolution as 7500, collision energy as 10/30/60 in NCE mode, spray voltage as 4 kV (positive) or −3.8 kV (negative), respectively.

#### 4.15.3 Data preprocessing and annotation

The raw data files were converted to files in mzXML format using ProteoWizard's "msconvert" program. Peak detection, extraction, alignment, and integration were performed using the CentWave algorithm in XCMS. The minfrac for annotation was set at 0.5, whereas the cutoff for annotation was set at 0.3. Lipid identification was carried out using the LipidBlast library, which utilized a spectral match and was developed using R and XCMS. Bioinformatic analysis was performed using the OmicStudio tools at https://www.omicstudio.cn/tool.

### 4.16 Statistics and reproducibility

All data are expressed as the mean ± SEM. Unpaired, two‐tailed Student's *t*‐test was performed to determine significant differences between two means. When comparing multiple groups, one‐way analysis of variance was used, followed by correction for multiple comparisons. To ensure the reproducibility of data, at least three independent experiments were conducted to verify the results. Statistical analysis was performed using GraphPad Prism 8.0 (GraphPad Prism Software). Significance was set at a *p* value < 0.05.

## AUTHOR CONTRIBUTIONS

N. L. and WJ. L. designed this research; N. L., L. H., H. X., and XY. H.; performed the research; J. C., WJ. X., YX. W., HQ. W., S. W., and H. Z. conducted parts of the experiments; N. L. analyzed the data and wrote the manuscript; YZ. X., WJ. L., and S. G. edited the manuscript. All authors have reviewed and approved the final manuscript.

## CONFLICT OF INTEREST STATEMENT

The authors declare no competing interest.

## ETHICS STATEMENT

All included patients provided informed consent, and the Biomedical Ethics Committee of the Anhui Medical University approved this study (Approved No. 83230239). The animal study was approved by the Experimental Animal Ethics Committee at the Anhui Medical University (LLSC20211170).

## Supporting information

Supporting information

Supporting information

Supporting information

Supporting information

## Data Availability

The data that support the findings of this study are available from the corresponding author upon reasonable request. The original, uncropped blot images can be found in Figure S8–S13.
