# Supplementary material for: Phosphatidylserine decarboxylase downregulation in uric acid‑induced hepatic mitochondrial dysfunction and apoptosis
Source: MedComm (2020). 2023 Jul 26;4(4):e336. doi: 10.1002/mco2.336 (PMC10369160; doi:10.1002/mco2.336)
Supplement: Supplementary file 3 — Supporting information [file MCO2-4-e336-s003.pdf]

Supplementary Information for

Phosphatidylserine decarboxylase downregulation in uric acid-induced hepatic mitochondrial dysfunction and apoptosis

Ning Liu[1,2,3,4, #], Lei Huang[1, #], Hu Xu[1, #], Xinyu He[1, #], Xueqing He[1], Jun Cao[1], Wenjun Xu[1], Yaoxing Wang[1], Hongquan Wei[1], Sheng Wang[5], Hong Zheng[1], Shan Gao [1, *], Youzhi Xu[1, *], Wenjie Lu[1, *]

[1] Basic Medical College, Anhui Medical University, Hefei 230032, Anhui, China.

[2] Zhejiang Provincial Laboratory of Life Sciences and Biomedicine, Key Laboratory of Growth Regulation and Transformation Research of Zhejiang Province, School of Life Sciences, Westlake University, Hangzhou 310024, Zhejiang, China.

[3] College of Life Sciences, Zhejiang University, Hangzhou, Zhejiang 310058, China.

[4] Institute of Biology, Westlake Institute for Advanced Study, Hangzhou 310024, Zhejiang Province, China.

[5] Center for Scientific Research, Anhui Medical University, Hefei 230032, Anhui, China.

[#] These authors contributed equally to this work.

[*] Corresponding author: Wenjie Lu (wenjie63136@163.com), Youzhi Xu (xuyouzhi@ahmu.edu.cn) and Shan Gao (aydgs@126.com). Address correspondence to: Dr. Wenjie Lu, Basic Medical College, Anhui Medical University, 81#, Mei Shan Road, Hefei 230032, China; E-mail: wenjie63136@163.com;

Table S1 Clinicopathological features of control subjects and hyperuricemia patients

Parameters	Control subjects	Hyperuricemia patients	p-Value
Sex	n=120	n=120	
Female	40(33%)	40(33%)	
Male	80(67%)	80(67%)	
Uric acid (μmol/L)	301.46±61.07	525.32±125.98	＜0.0001[a]
Age (year)	39.71±13.36	42.55±15.08	= 0.1236[a]
BMI (kg/m^2)	21±1.15	22.98±1.61	＜0.0001[a]
TC (mmol/L)	3.97±0.83	4.62±1.8	= 0.0004[a]
TG (mmol/L)	1.08±0.4	2.05±1.73	＜0.0001[a]
HDL-C (mmol/L)	1.46±0.27	1.12±0.35	＜0.0001[a]
n-HDL-C (mmol/L)	2.84±0.56	3.33±1.74	= 0.0035[a]
LDL-C (mmol/L)	1.95±0.82	2.59±1.46	＜0.0001[a]
VLDL (mmol/L)	0.39±0.11	0.74±0.63	＜0.0001[a]
APOA (g/L)	1.33±0.14	1.38±0.31	= 0.1366[a]
APOB (g/L)	0.7±0.19	0.84±0.27	＜0.0001[a]
LP(a) (mg/L)	183.78±73.31	258.72±208.11	= 0.0002[a]
LDH (U/L)	190.35±34.98	229.55±68.62	＜0.0001[a]
UREA (mmol/L)	4.5±0.82	8.26±5.41	＜0.0001[a]
CRE (μmol/L)	76.27±11.95	141.25±146.57	＜0.0001[a]
eGFR (ml/min/1.73m^2)	134.33±29.25	72.55±39.62	＜0.0001[a]
GLU (mmol/L)	5.15±0.56	6.06±1.29	＜0.0001[a]

[a] ***t*-Test.**

Abbreviations: TC, total cholesterol; TG, triglycerides; HDL-C, high-density lipoprotein cholesterol; n-HDL-C, non-high-density lipoprotein cholesterol; LDL-C, low-density lipoprotein cholesterol; VLDL-C, very low-density lipoprotein cholesterol; BMI, body mass index; APOA, apolipoprtein A; APOB, apolipoprtein B; LP(a), lipoprotein(a); LDH, lactate dehydrogenase; UREA, urea; CRE, creatinine; eGFR, calculated glomerular filtration rate; GLU, glucose**.**

Table S2 Primers sequences used for quantitative RT-PCR

Gene	Sequence (forward)	Sequence (reverse)
**Mouse**		
Pisd	5′ TCAGTCAGAGAAGCAGCCAGGAC	5′ CAGGAGGTAGTGGAGGATGGTCAG
PTDSS1	5′ CTTCTTTCTCATCATCAGCGTG	5′ GGATTTAACCTGCTCGAAGTTC
PTDSS2	5′ CTTTGTTCCTGCACACTTCATT	5′ CACACTGATGATCATGCACATC
GAPDH	5′ GGTTGTCTCCTGCGACTTCA	5′ TGGTCCAGGGTTTCTTACTCC
**Human**		
Pisd	5′ CTTTGTACAAGTCAGTGCCAAC	5′ CCAGATGTACAGGCTGTAGAC
GAPDH	5′ CAGGAGGCATTGCTGATGAT	5′ GAAGGCTGGGGCTCATTT

Supplementary figures and figure legends

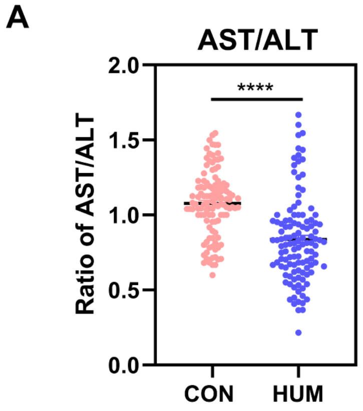

Figure S1. Ratio of AST to ALT in hyperuricemia patients. (A) The ratio of serum AST to ALT in subjects with normal UA levels and patients with hyperuricemia. Data are presented as means ± SEM. ****$P < 0.0001$.

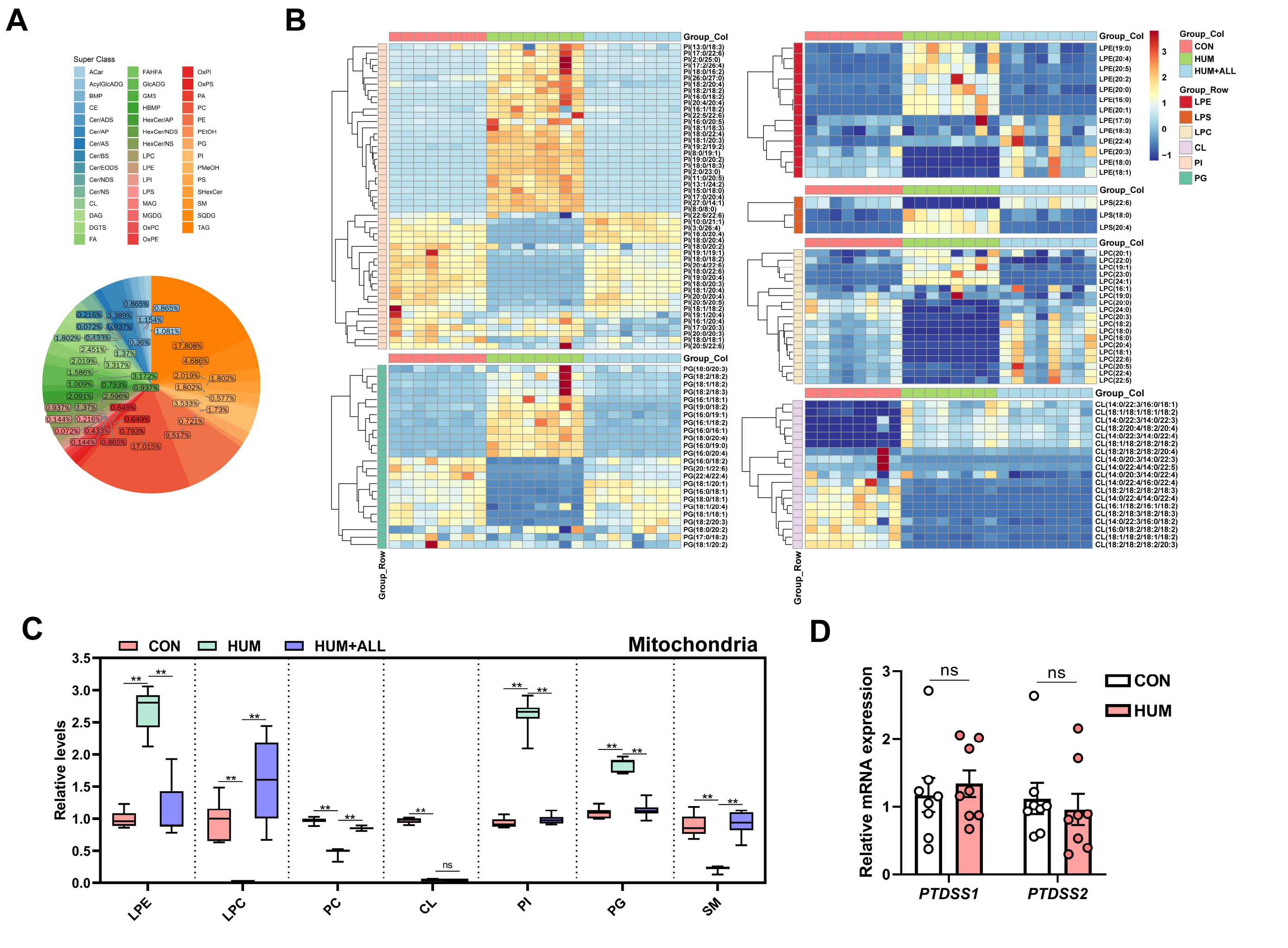

Figure S2. Proportion and content of various lipids in liver mitochondria of hyperuricemia model mice. (A) The proportion of various lipids in mouse liver mitochondria. (B) Heatmap of the number of differentially abundant LPEs, LPSs, LPCs, CLs, PIs, and PGs in mouse liver mitochondria of CON (n = 8), HUM (n = 8), and HUM+ALL (n = 8) mice. (C) Total LPE, LPC, PC, CL, PI, PG, SM were quantified in mitochondria of CON group, HUM group, and HUM+ALL group. (D) *PTDSS1*, *PTDSS2* mRNA levels were determined via real-time PCR of mouse liver tissues. Data are presented as means ± SEM. ** $P < 0.01$, * P

< 0.05, ns indicates no significance.

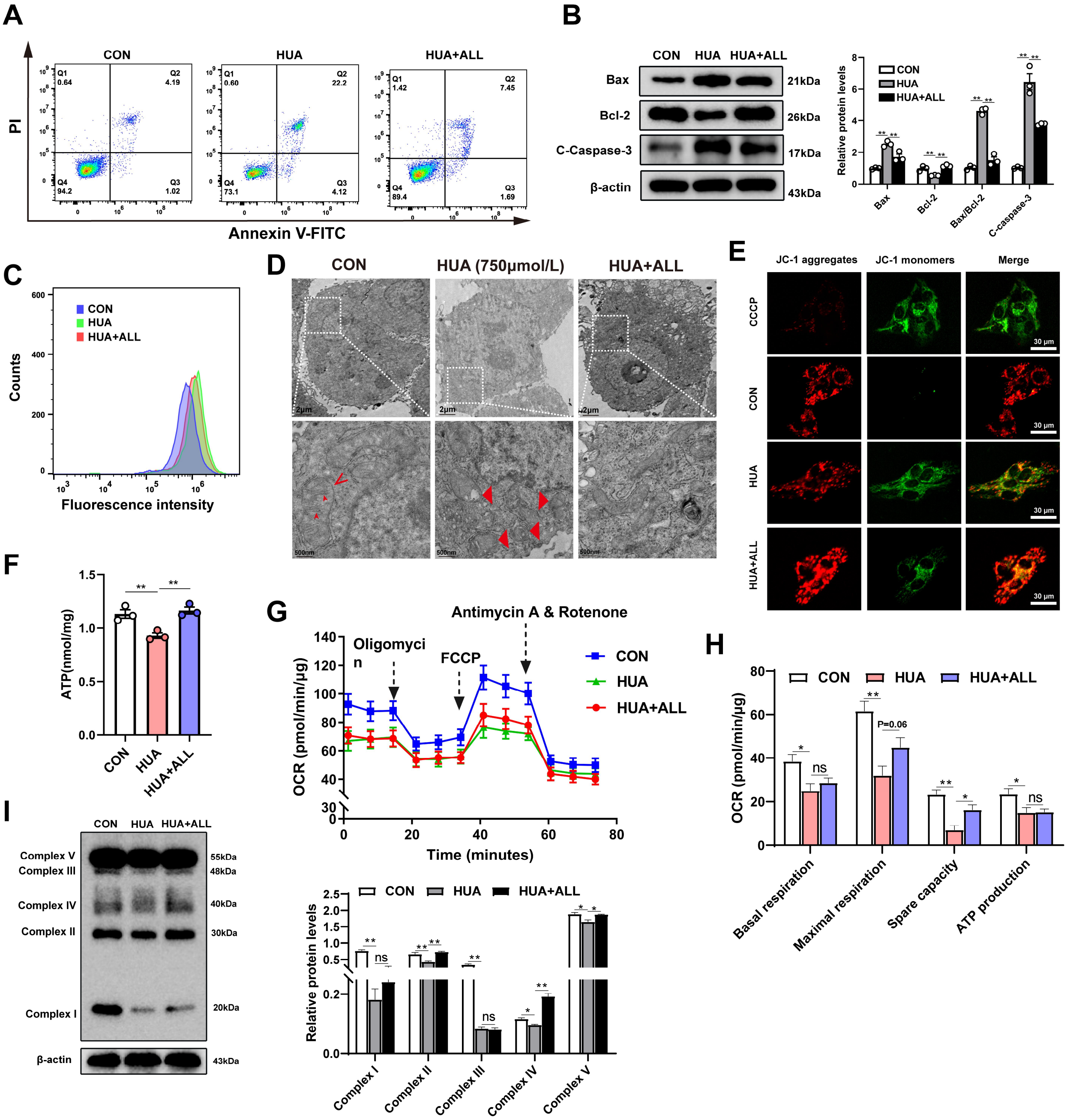

Figure S3. UA causes mitochondrial dysfunction and apoptosis in HepG2 cells. (A) UA-induced apoptosis in HepG2 cells. The apoptosis was detected by Annexin V-FITC and PI staining. (B) Bcl-2, Bax and Cleaved caspase-3 protein levels of three groups in total protein were detected by western blot analysis. (C) The levels of ROS in L02 cells treated with UA (750 μmol/L) or allopurinol (100 μmol/L) were detected by flow cytometry. (D) Transmission electron microscopy images at original magnifications of ×3000 (top) and ×10,000 (bottom). Electron microscopy representative images demonstrated marked alterations in mitochondrial morphology in HepG2 cells incubated with 750 μmol/L UA for 48 h. Higher magnification (×10,000) revealed a disruption of mitochondrial double membrane (thick arrowhead "◀") in UA-treated cells compared with well-preserved cristae (thin arrowhead "‣") and double membrane (thin arrow "<") in control cells. (E) The changes of MMP were measured by JC-1 fluorescence method in HepG2 cells treated with UA or allopurinol. (F) ATP content was detected in three groups. (G) Mitochondrial respiration measurements of OCR were performed with a Seahorse metabolic analyzer. Oligomycin (1.5 μM), FCCP (1 μM), and rotenone (0.5 μM) combined with antimycin (0.5 μM) were added sequentially to HepG2 cells treated with or without UA (750 μmol/L) and allopurinol (100 μmol/L). (H) Quantitative analysis of mitochondrial function parameters (basal respiration, maximal respiration, spare capacity, and ATP

production) was shown in the bar charts (n=5). (I) OXPHOS proteins expression (Complex I-V) of HepG2 cells stimulated high UA or treated with allopurinol were analyzed by western blotting. Data are means ± SEM. ** $P < 0.01$, * $P < 0.05$, ns means no significance.

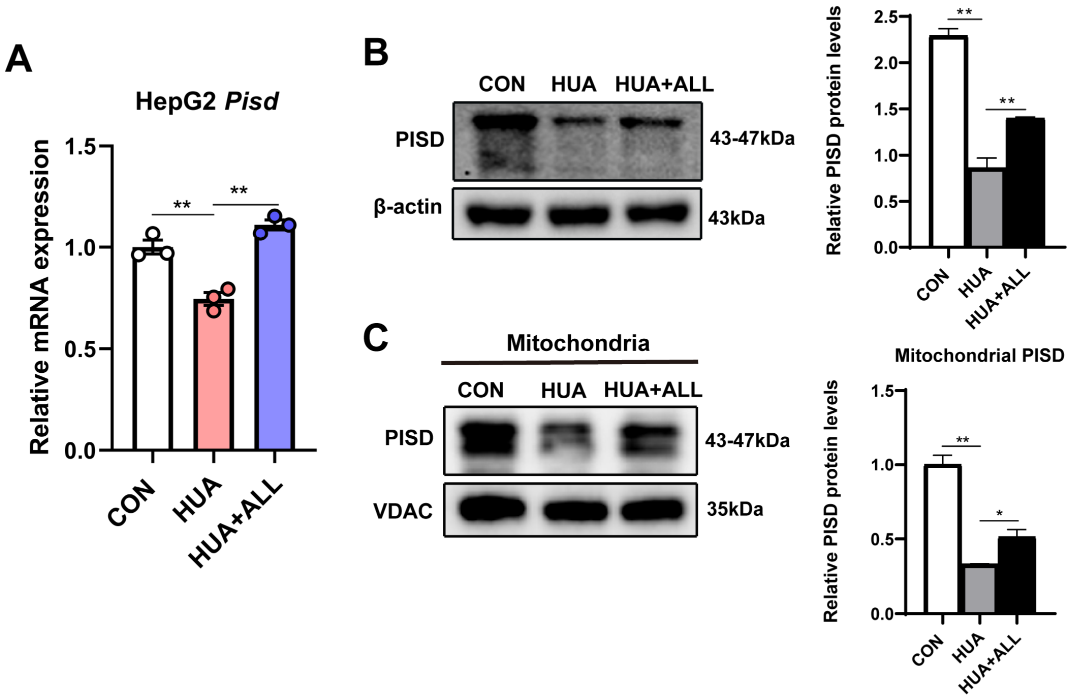

Figure S4. The expression of PISD is inhibited by high uric acid in HepG2 cells. (A) *Pisd* mRNA levels were determined by real-time PCR in HepG2 cells of three groups. (B) Representative western blots showing protein expression of PISD in HepG2 cells among three groups. (C) PISD protein levels in mitochondria of three groups. Band intensities of each protein were quantified using ImageJ and normalized to that of β-actin or VDAC band. Data are means ± SEM. ** $P < 0.01$, * $P < 0.05$.

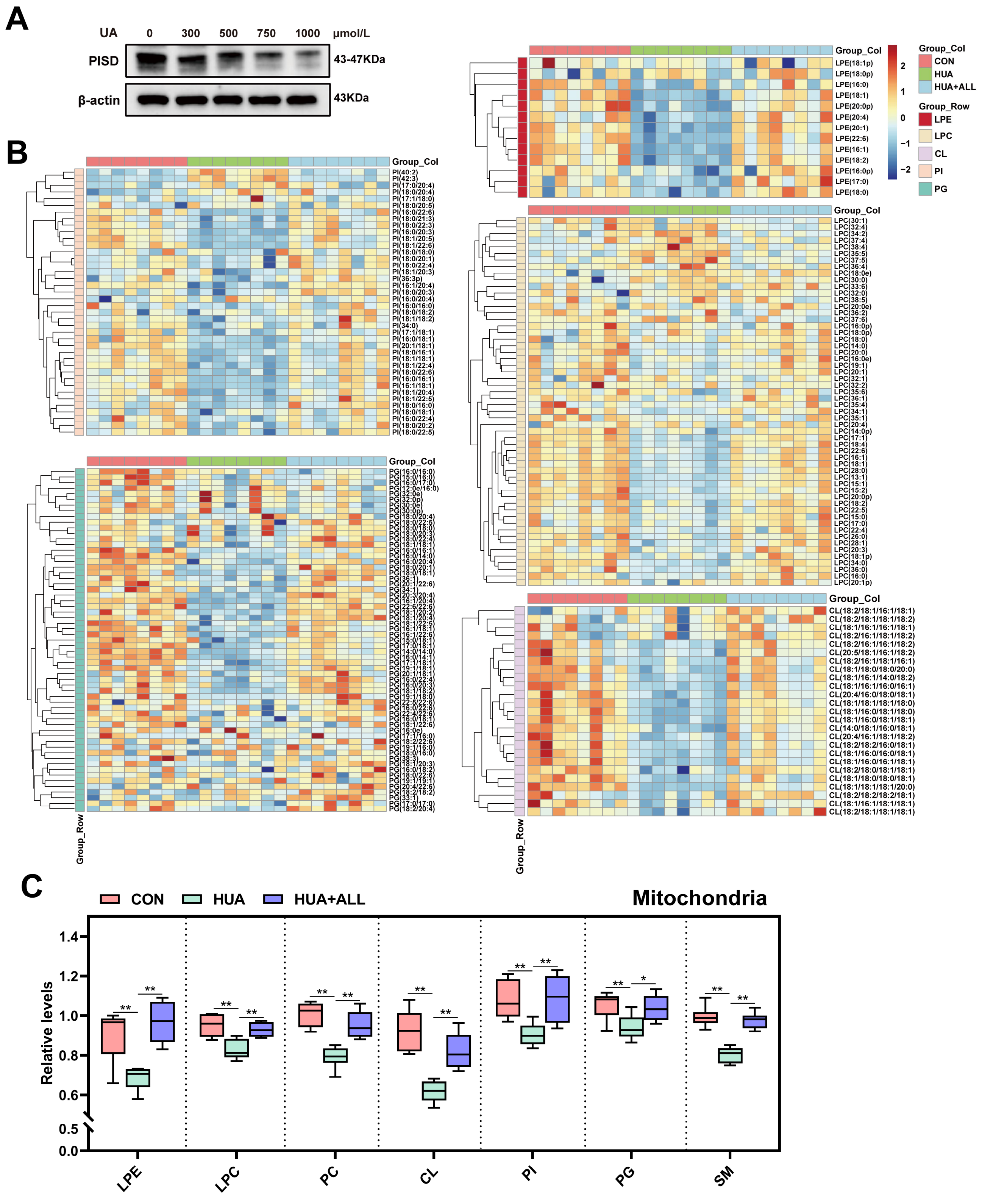

Figure S5. The expression of PISD and content of various lipids in mitochondria of L02 cells incubated with high UA. (A) L02 cells were exposed to different UA concentrations of 0, 300 μmol/L, 500 μmol/L, 750 μmol/L, 1000 μmol/L for 48h. PISD protein levels were detected by western blotting. (B) Heatmap of the abundance LPEs，LPCs, CLs, PIs, and PGs in L02 cells mitochondria of CON (n = 8), HUA (n = 8), and HUA+ALL (n = 8) group. (C) Total LPE, LPC, PC, CL, PI, PG, SM were quantified in L02 cells mitochondria of CON group, HUA group, and HUA+ALL group. Data are presented as means ± SEM. ** *P* < 0.01, * *P* < 0.05.

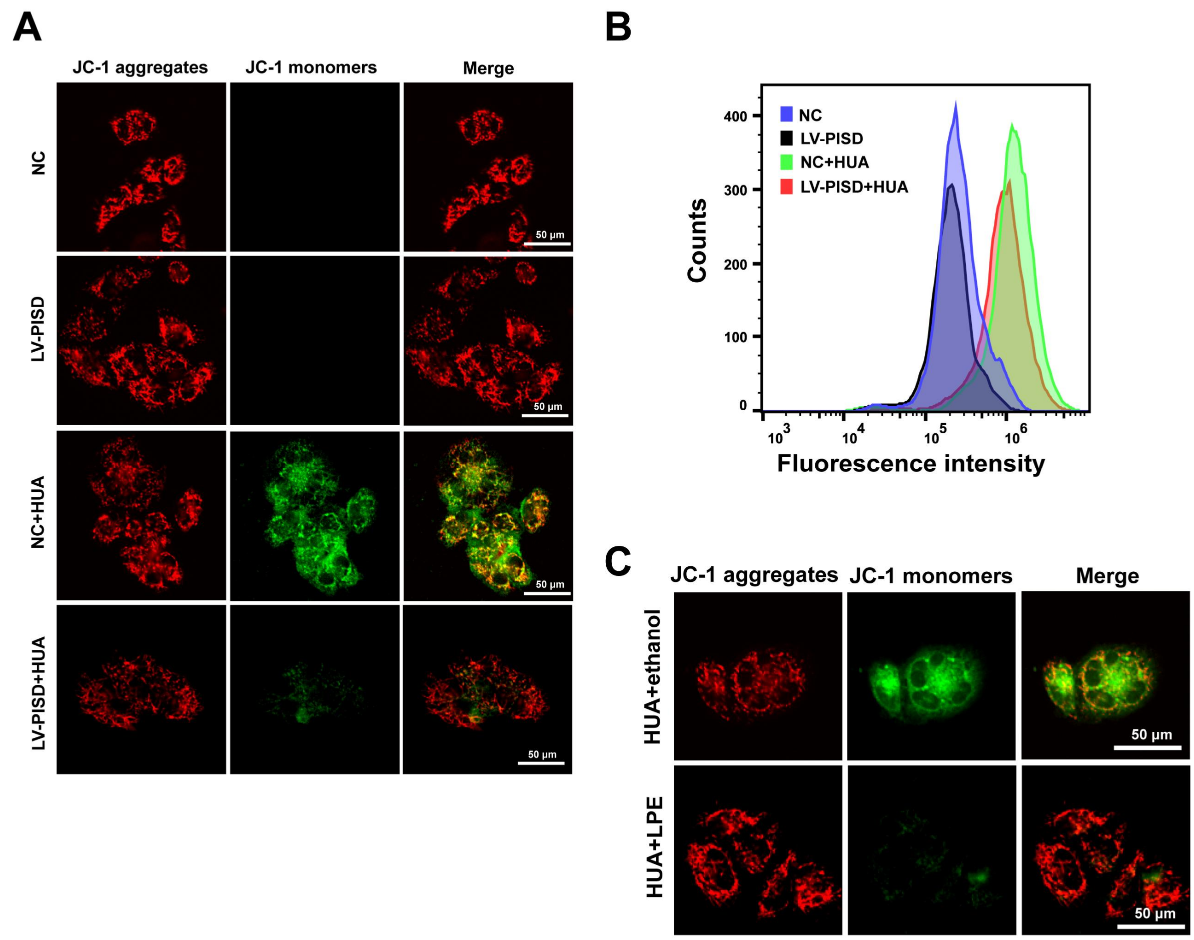

Figure S6. Changes in MMP, the levels of ROS, and apoptosis in L02 cells overexpressing PISD and supplemented with LPE. (A) Changes in MMP in L02 cells of four groups (NC group, LV-PISD group, NC+HUA group, LV-PISD+HUA group). (B) The levels of ROS in L02 cells of NC group, LV-PISD group, NC+HUA group, LV-PISD+HUA group. (C) Changes in MMP in the HUA+ethanol and HUA+LPE group.

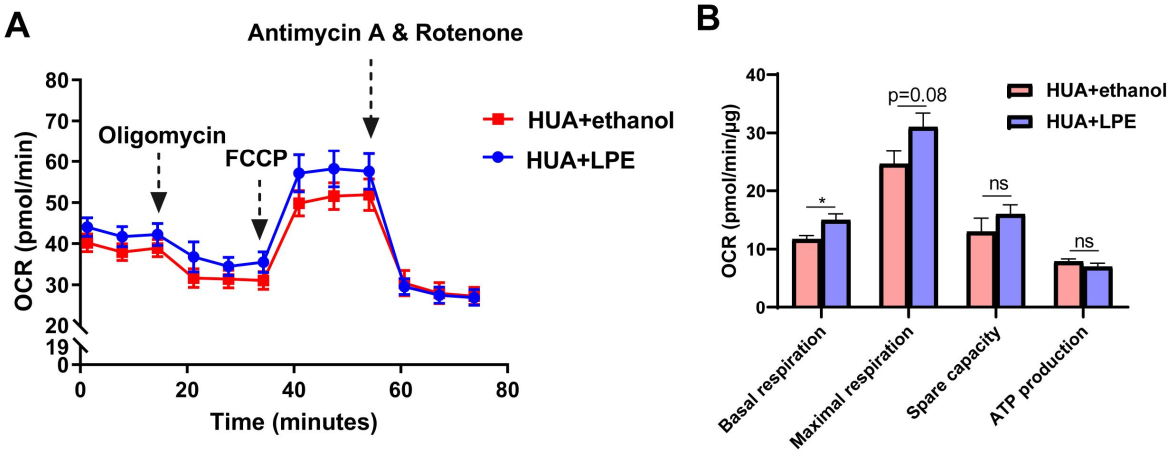

Figure S7. OCR of HepG2 was measured after lyso-PE supplementation. (A) Mitochondrial respiration measurements of OCR were performed with a Seahorse metabolic analyzer. Oligomycin (1.5 μM), FCCP (1 μM), and rotenone (0.5 μM) combined with antimycin (0.5 μM) were added sequentially to HepG2 cells in HUA+ethanol group and HUA+LPE group. (B) Quantitative analysis of mitochondrial function parameters (basal respiration, maximal respiration, spare capacity, and ATP production) was shown in the bar charts (n = 5). Data are means ± SEM. ** $P < 0.01$, * $P < 0.05$, ns means no significance.

Fig.1F

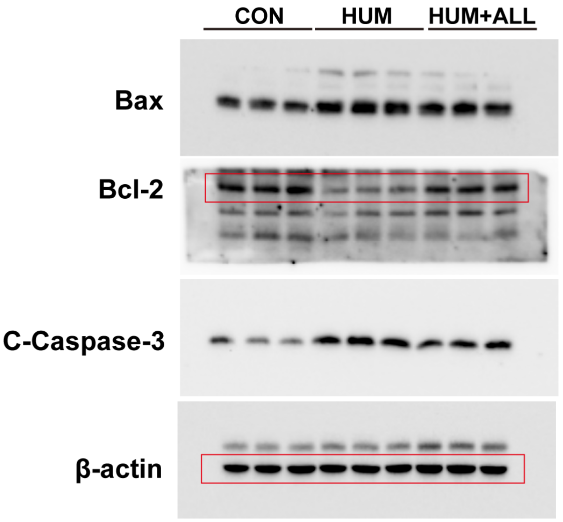

Figure S8. Original images for immunoblots shown in Figure 1

Fig.2H

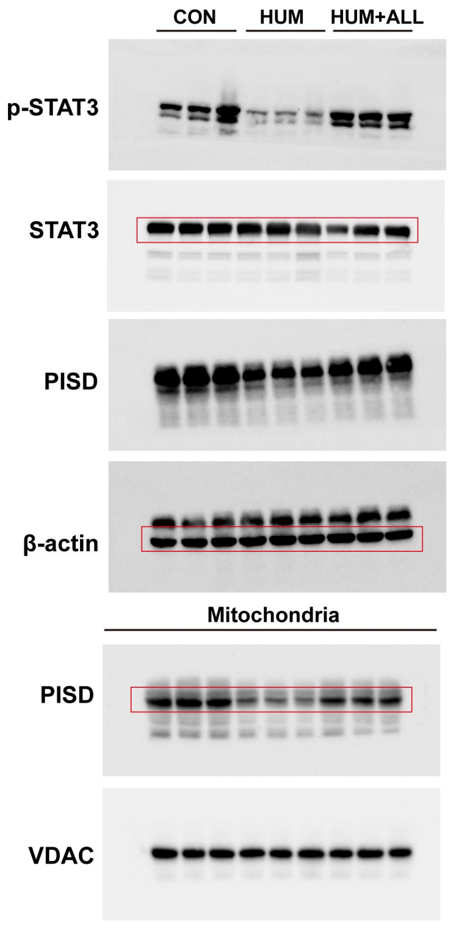

Fig.2K

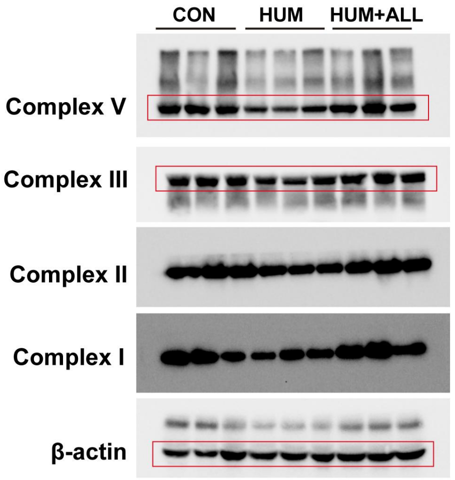

Figure S9. Original images for immunoblots shown in Figure 2

Fig.3B

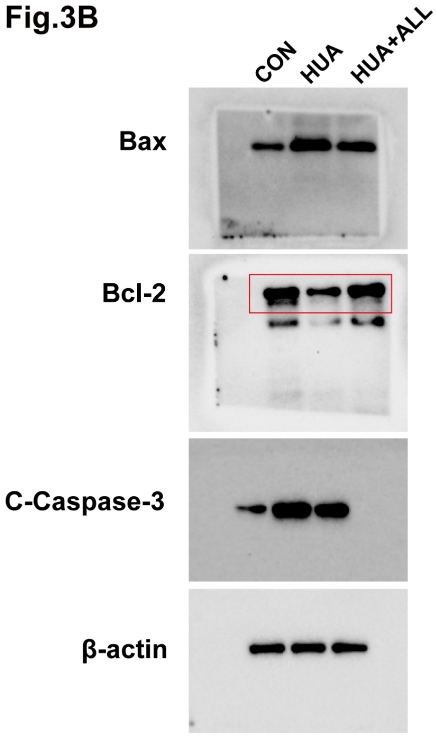

Fig.3I

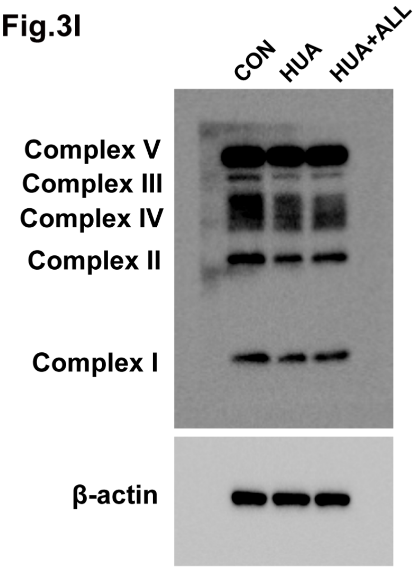

Figure S10. Original images for immunoblots shown in Figure 3

Fig.4B

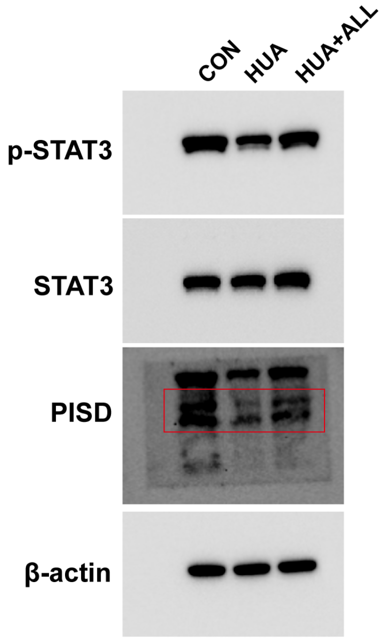

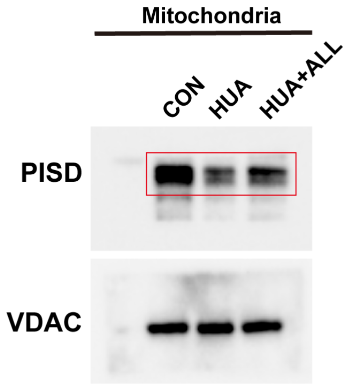

Figure S11. Original images for immunoblots shown in Figure 4

Fig.5B

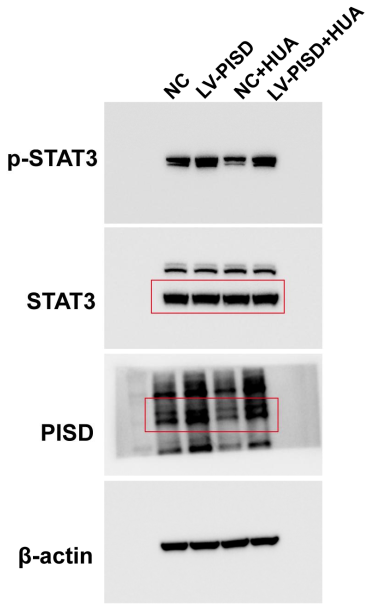

Fig.5D

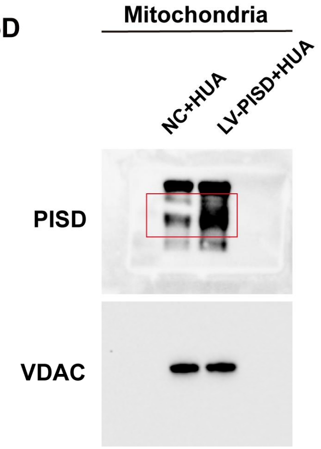

Fig.5L

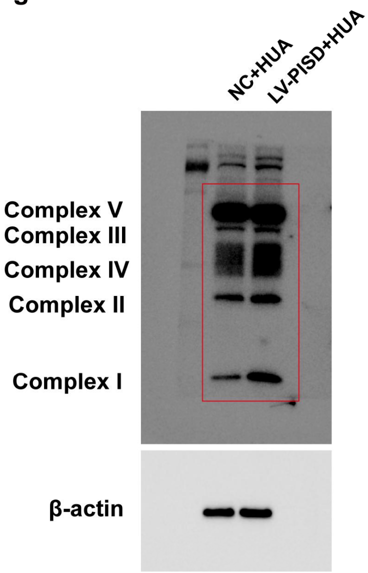

Fig.5N

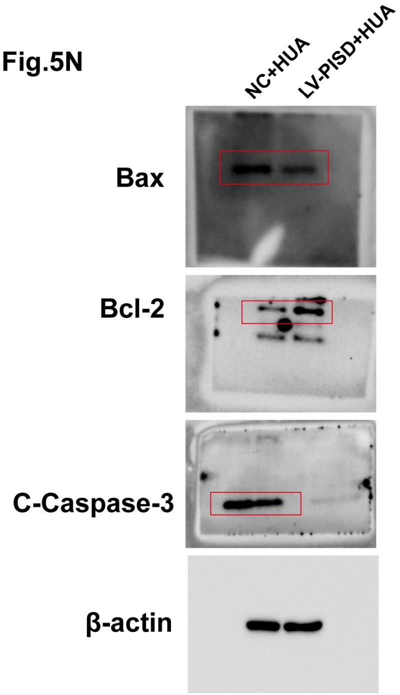

Figure S12. Original images for immunoblots shown in Figure 5

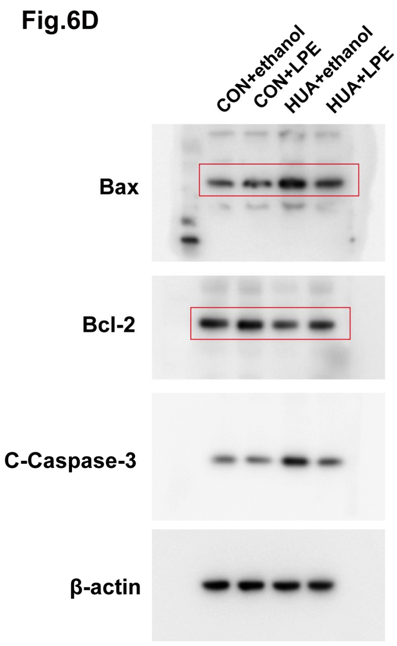

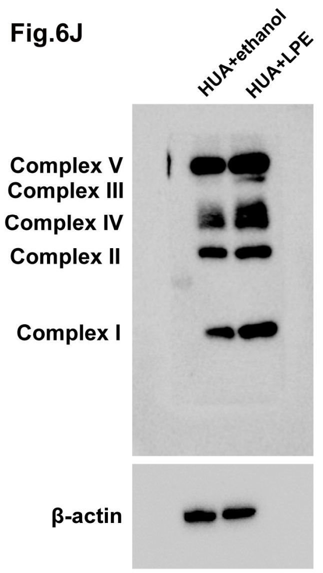

Figure S13. Original images for immunoblots shown in Figure 6
