# Supplementary material for: Phosphatidylserine decarboxylase downregulation in uric acid‑induced hepatic mitochondrial dysfunction and apoptosis
Source: MedComm (2020). 2023 Jul 26;4(4):e336. doi: 10.1002/mco2.336 (PMC10369160; doi:10.1002/mco2.336)
Supplement: Supplementary file 4 — Supporting information [file MCO2-4-e336-s001.docx]

**Description of Additional Supplementary Files**

**File name:** Supplementary Movie S1

**Description:** Observation of cell proliferation in L02 cells supplemented with LPE by living cell station (Celldiscoverer 7.0, Carle Zeiss). After stimulating L02 cells with UA + LPE, we placed the cell plate into the living cell growth detection instrument. Three points were selected in each well of the cell for monitoring and the image of each point was captured every 2 h, with a total observation period of 48 h; the cell plate was not moved during this time.

**File name:** Supplementary Movie S2

Description: Observation of the movement of lipid droplets in L02 cells supplemented with UA+ethanol by living cell station.
